# Evolutionary characterisation of lung adenocarcinoma morphology in TRACERx

**DOI:** 10.1038/s41591-023-02230-w

**Published:** 2023-04-12

**Authors:** Takahiro Karasaki, David A. Moore, Selvaraju Veeriah, Cristina Naceur-Lombardelli, Antonia Toncheva, Neil Magno, Sophia Ward, Maise Al Bakir, Thomas B.K. Watkins, Kristiana Grigoriadis, Ariana Huebner, Mark S. Hill, Alexander M. Frankell, Christopher Abbosh, Francisco Gimeno-Valiente, Sadegh Saghafinia, Nnennaya Kanu, Michelle Dietzen, Oriol Pich, Emilia Lim, Carlos Martinez-Ruiz, James R.M. Black, Clare Puttick, Dhruva Biswas, Brittany B. Campbell, Claudia Lee, Emma Colliver, Katey S.S. Enfield, Sonya Hessey, Crispin T. Hiley, Simone Zaccaria, Kevin Litchfield, Nicolai J. Birkbak, Elizabeth Larose Cadieux, Jonas Demeulemeester, Peter Van Loo, Prasad S. Adusumilli, Kay See Tan, Waseem Cheema, Francisco Sanchez-Vega, David R. Jones, Natasha Rekhtman, William D. Travis, Allan Hackshaw, TRACERx Consortium, Teresa Marafioti, Roberto Salgado, John Le Quesne, Andrew G. Nicholson, Nicholas McGranahan, Charles Swanton, Mariam Jamal-Hanjani, Mariam Jamal-Hanjani, Nicholas McGranahan, Charles Swanton, Nicholas McGranahan, Nicolai J. Birkbak, Kevin Litchfield, Simone Zaccaria, Charles Swanton, Takahiro Karasaki, David A. Moore, Maise Al Bakir, Brittany B. Campbell, Christopher Abbosh, Sonya Hessey, Crispin T. Hiley, Mariam Jamal-Hanjani, Charles Swanton, Allan Hackshaw, UCL CTC (Cancer Trials Centre), David A. Moore, Andrew G. Nicholson, John Le Quesne, Roberto Salgado, Maise Al Bakir, Takahiro Karasaki, Selvaraju Veeriah, Cristina Naceur-Lombardelli, Antonia Toncheva, Neil Magno, EHP STP (Experimental Histopathology Science Technology Platform), Sophia Ward, ASF STP (Advanced Sequencing Facility Science Technology Platform), Takahiro Karasaki, David A. Moore, Michelle Dietzen, Takahiro Karasaki, Kristiana Grigoriadis, Alexander M. Frankell, Thomas B.K. Watkins, Emilia Lim, Mark S. Hill, Brittany B. Campbell, Takahiro Karasaki, Carlos Martinez-Ruiz, James R.M. Black, Clare Puttick, Dhruva Biswas, Francisco Gimeno-Valiente, Sadegh Saghafinia, Nnennaya Kanu, Takahiro Karasaki, Ariana Huebner, Maise Al Bakir, Christopher Abbosh, Alexander M. Frankell, Takahiro Karasaki, Nicolai J. Birkbak, Charles Swanton, Takahiro Karasaki, Clare Puttick, Oriol Pich, Clare Puttick, Claudia Lee, Kristiana Grigoriadis, Emma Colliver, Katey S.S. Enfield, Crispin T. Hiley, Nicholas McGranahan, Charles Swanton, Takahiro Karasaki, Mark S. Hill, Kevin Litchfield, Allan Hackshaw, Prasad S. Adusumilli, Kay See Tan, Waseem Cheema, Francisco Sanchez-Vega, David R. Jones, Natasha Rekhtman, William D. Travis, David A. Moore, Takahiro Karasaki, Thomas B.K. Watkins, Alexander M. Frankell, Kristiana Grigoriadis, Emma Colliver, Katey S.S. Enfield, Elizabeth Larose Cadieux, Jonas Demeulemeester, Peter Van Loo, Francisco Sanchez-Vega, David R. Jones, Natasha Rekhtman, William D. Travis, Teresa Marafioti, Roberto Salgado, John Le Quesne, Andrew G. Nicholson, Nicholas McGranahan, Mariam Jamal-Hanjani, Charles Swanton

**Affiliations:** Study Design, Conduct and Clinical and Laboratory Oversight; Informatics Supervision; Cohort and Clinical Annotation; Central Pathology Review; Sample Extraction and Management; Cohort Overview; Genetic Alteration and Evolutionary Dependency; Transcriptomic Data Analysis; Metastasis Data Analysis; Circulating Tumour DNA Data Analysis; Tumour Immune Microenvironment Analysis; Regression Analysis, Survival analysis and Statistics; External cohort data analysis; Manuscript Writing; 1 Cancer Research UK Lung Cancer Centre of Excellence, University College London Cancer Institute, London, UK; 2 Cancer Evolution and Genome Instability Laboratory, The Francis Crick Institute, London, UK; 3 Department of Cellular Pathology, University College London Hospitals, London, UK; 4 Advanced Sequencing Facility, The Francis Crick Institute, London, UK; 5 Cancer Genome Evolution Research Group, Cancer Research UK Lung Cancer Centre of Excellence, University College London Cancer Institute, London, UK; 6 Bill Lyons Informatics Centre, University College London Cancer Institute, London, UK; 7 Program in Genetics and Genome Biology, The Hospital for Sick Children, Toronto, Ontario, Canada; 8 The Arthur and Sonia Labatt Brain Tumour Research Centre, The Hospital for Sick Children, Toronto, Ontario, Canada; 9 Cancer Metastasis Lab, University College London Cancer Institute, London, UK; 10 Computational Cancer Genomics Research Group, University College London Cancer Institute, London, UK; 11 The Tumour Immunogenomics and Immunosurveillance (TIGI) Lab, University College London Cancer Institute, London, UK; 12 Department of Molecular Medicine, Aarhus University Hospital, Aarhus, Denmark; 13 Department of Clinical Medicine, Aarhus University, Aarhus, Denmark; 14 Bioinformatics Research Center, Aarhus University, Aarhus, Denmark; 15 Cancer Genomics laboratory, The Francis Crick Institute, London, UK; 16 Medical Genomics, University College London Cancer Institute, London, UK; 17 Department of Human Genetics, KU Leuven, B-3000 Leuven, Belgium; 18 Thoracic Service, Department of Surgery, Memorial Sloan Kettering Cancer Center, NY, USA; 19 Center for Cell Engineering, Memorial Sloan Kettering Cancer Center, NY, USA; 20 Department of Epidemiology and Biostatistics, Memorial Sloan Kettering Cancer Center, NY, USA; 21 Department of Pathology, Memorial Sloan Kettering Cancer Center, NY, USA; 22 Cancer Research UK and UCL Cancer Trials Centre, London, UK; 23 Department of Pathology, GZA/ZNA Hospitals, Antwerp, Belgium; 24 Division of Research, Peter Mac Callum Cancer Centre, Melbourne, Australia; 25 Cancer Research UK Beatson Institute, Glasgow, UK; 26 Institute of Cancer Sciences, University of Glasgow, Glasgow, UK; 27 Department of Histopathology, Royal Brompton and Harefield Hospitals, Guy’s and St Thomas’ NHS Foundation Trust, London, UK; 28 National Heart and Lung Institute, Imperial College, London, UK; 29 Department of Medical Oncology, University College London Hospitals, London, UK

## Abstract

Lung adenocarcinomas (LUADs) display a broad histological spectrum from low-grade lepidic tumours through to mid-grade acinar and papillary and high-grade solid, cribriform and micropapillary tumours. Little is known as to how morphology reflects tumour evolutionary history and disease progression. Whole exome sequencing data generated from 805 primary tumour regions and 121 paired metastatic tumours across 248 LUADs from the TRACERx 421 cohort, as well as RNA-Seq data from 463 primary tumour regions, were integrated with detailed histopathological analysis of tumour morphology at the whole and regional tumour level. Tumours with predominantly high-grade architectural patterns showed increased chromosomal complexity, with higher levels of loss of heterozygosity (LOH) and subclonal somatic copy number alterations (SCNAs). Individual regions in predominantly high-grade pattern tumours tended to be highly proliferative and less clonally diverse, potentially reflective of large recent subclonal expansions. Co-occurence of truncal loss/LOH of chromosome 3p and 3q was enriched in predominantly low/mid-grade tumours, whilst purely undifferentiated solid pattern tumours had a higher frequency of truncal 3q gains and *SMARCA4* gene alterations compared with other subtypes, including mixed solid pattern tumours, suggesting distinct evolutionary trajectories. Clonal evolution analysis revealed that tumours tend to evolve towards higher grade patterns. The presence of micropapillary pattern and ‘spread through air spaces’ (STAS) were associated with an increased risk of intra-thoracic-only recurrence, in contrast to the presence of solid/cribriform patterns, necrosis, and pre-operative circulating tumour DNA (ctDNA) detection, which were associated with increased risk of extra-thoracic recurrence. Overall, these data provide insights into the relationship between LUAD histological subtypes and their underlying evolutionary genomic landscape, as well as clinical risk and clonal nature of metastatic dissemination.

## Introduction

**Extended Data Fig. 1 |:**
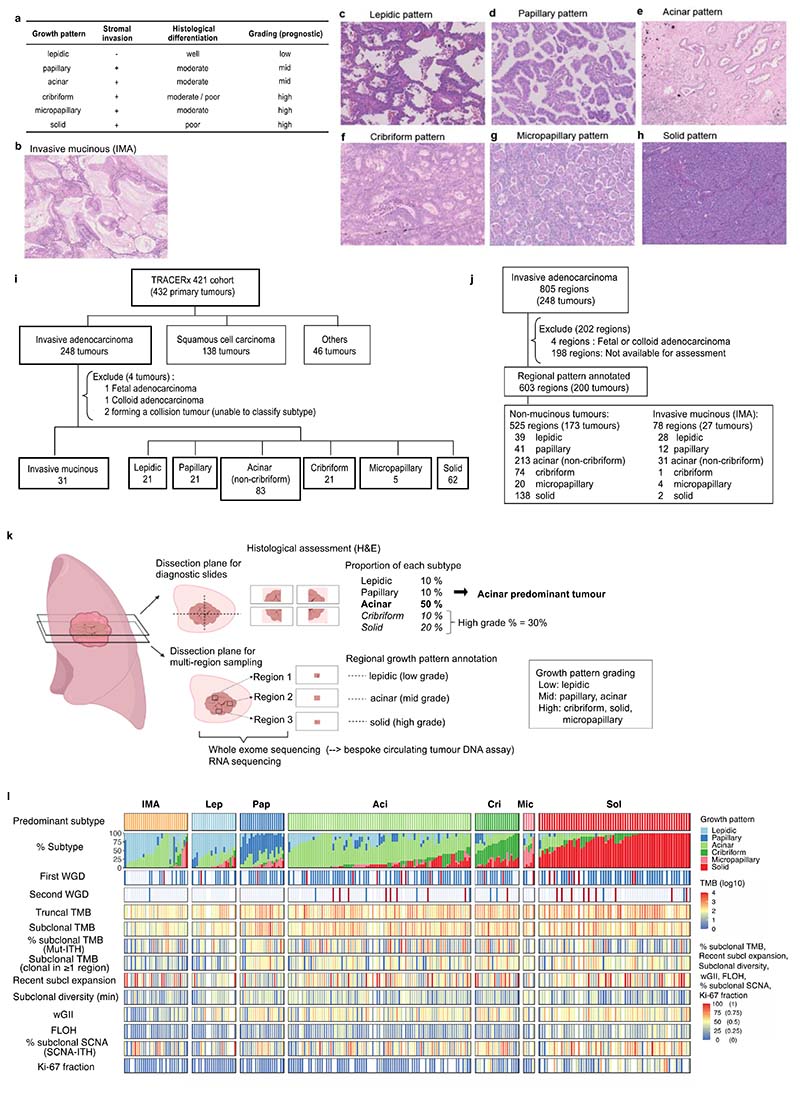
Histopathological assessment of the TRACERx 421 LUAD cohort **a.** Definition and categorisation of LUAD growth patterns. **b.** Representative haematoxylin and eosin (H&E) photo of invasive mucinous adenocarcinoma (IMA). **c-h.** Representative H&E photos of lepidic **(c)**, papillary **(d)**, acinar **(e)**, cribriform **(f)**, micropapillary **(g)**, and solid **(h)** pattern observed in LUAD. **i.** Number of each predominant subtype tumour in the TRACERx 421 cohort. **j.** Number of regions with growth pattern assessment. **k.** Schematic of histological assessment in the TRACERx study. Proportions of each subtype in the diagnostic slides were reported, and the predominant subtype was used to label each tumour. Multi-region sampling specimens were processed for whole exome sequencing, and each region was annotated with the representative growth pattern. **l**. Overview of TRACERx 421 LUAD cohort including invasive mucinous adenocarcinoma. Each column represents one tumour (n = 244). The proportion of each growth pattern based on diagnostic sectional area, genomic variables, and Ki-67 fraction by immunohistochemical staining are summarised. WGD, whole genome doubling; TMB, tumour mutational burden; ITH, intra-tumour heterogeneity; Mut, mutational; wGII, weighted genome instability index; FLOH, fraction of the genome subject to loss of heterozygosity; SCNA, somatic copy number alteration.

Lung cancer, the leading cause of cancer-related death globally, encompasses a range of different histological subtypes. Lung adenocarcinoma (LUAD), the most common histological entity, is a morphologically and genetically diverse disease with various histological features which relate to tumour behaviour. This includes the range of tissue architectural growth patterns from ‘lepidic’, which is well-differentiated and non-invasive, through to undifferentiated ‘solid’^1^ (Extended Data Fig. 1a-h), as well as mucinous differentiation observed in invasive mucinous adenocarcinoma (IMA)^2^, the presence of ‘spread through air spaces’ (STAS)^3^, and the density of tumour infiltrating lymphocytes (TILs)^4^. Growth patterns, categorised as lepidic (low-grade), papillary and acinar (mid-grade), and cribriform, micropapillary, and solid (high-grade), are frequently mixed within a single LUAD tumour, and the proportion of high-grade patterns within each tumour is known to impact patient outcome^5^. However, the biological implications of different growth patterns, and their potential genomic underpinnings, are poorly understood.

Previously published genomic analysis of single tumour regions has shown that high-grade tumours harbour increased tumour mutational burden (TMB) and rates of whole genome doubling (WGD), as well as APOBEC-related mutagenesis in comparison to other subtypes^6^. Rates of somatic alterations in specific genes, such as *TP53*, together with the fraction of the genome altered by somatic copy number alterations (SCNAs), have been found to be increased in tumours with predominantly high-grade morphological patterns^6^.

Individual LUADs can display a range of growth patterns and detailed multi-region analysis has the potential to identify determinants of the transition from low- to high-grade patterns. However, the evolutionary features which determine histological subtypes and potential genomic and transcriptomic drivers of high-grade disease have not been well established. In a previous study of 10 LUADs with microdissected multi-region omics data, differences in transcriptomic profiles were more strongly associated with a shift between growth patterns rather than genomic alterations^7^. We hypothesised that multi-region tumour sampling data from the TRACERx (TRAcking non–small cell lung Cancer Evolution through therapy (Rx)) cohort could shed further light on the evolutionary progression of these distinct histological LUAD subtypes. In particular, the presence of distinct growth patterns within individual tumours provides a natural experiment to explore which genomic features relate to specific growth patterns, while controlling for background clinical and germline features.

TRACERx is a prospective observational study of non-small cell lung cancer (NSCLC) from diagnosis through to cure or relapse, with multi-region primary and metastatic tumour sampling alongside longitudinal circulating tumour DNA (ctDNA) analysis and detailed clinical annotation, including data relating to outcomes^8,9^. The TRACERx 421 cohort represents the first 421 patients prospectively recruited into the study^10,11^ in which 248 primary LUADs (805 tumour regions) from 242 patients and 121 paired metastatic lymph nodes and recurrence tumours from 65 patients with centralised pathologic review and subtyping were included in this analysis (Extended Data Fig. 1i-j). RNA-Seq data were available for 189 primary tumours (463 tumour regions). Here we describe the molecular characteristics of LUAD evolution and progression with respect to tumour morphology using multi-region whole exome sequencing and transcriptomic data.

## Results

### Clonal evolutionary characteristics of LUAD growth patterns

**Fig. 1 |:**
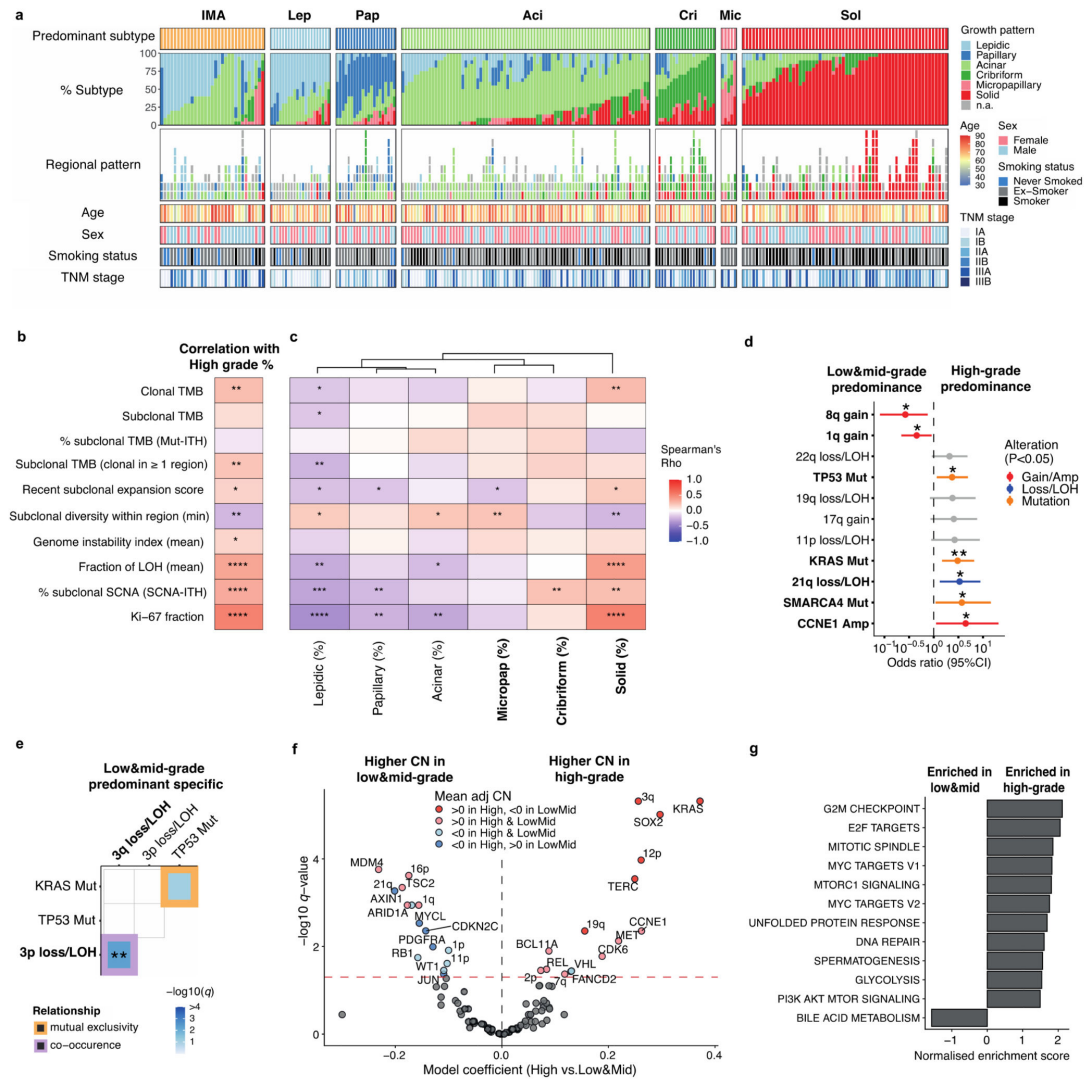
Determinants of inter-tumoural growth pattern heterogeneity a. Overview of TRACERx 421 LUAD cohort (n = 244 tumours). Each column represents one tumour. Invasive mucinous adenocarcinomas (IMAs) were included. Fetal adenocarcinoma, colloid adenocarcinoma, and two tumours from a collision tumour determined by genomic analysis were excluded from the analysis. The proportion of each growth pattern based on diagnostic sectional area, the growth pattern per region, and basic clinical information are summarised. **b-c.** Correlation of genomic variables and (**b**) proportion of high-grade patterns and (**c**) proportion of each growth pattern within each tumour, with high-grade patterns indicated in bold. Colour scale reflects Spearman’s rank correlation coefficient (rho). Correlation *P* values were corrected for multiple testing according to Benjamini-Hochberg (BH) and and asterisks indicate *q* value ranges * *q* < 0.05, ** *q* <0.01, *** *q* < 0.001, **** *q* < 0.0001. TMB, tumour mutational burden; ITH, intra-tumour heterogeneity; LOH, loss of heterozygosity; SCNA, somatic copy number alteration. **d.** Adjusted odds ratios (OR) of truncal genomic alterations associated with the predominantly high-grade pattern tumours. Genomic alterations selected by the model simplification are shown, with statistically significant alterations indicated in bold. The OR and *P* values (type II ANOVA) in the figure come from single multivariable logistic regression analysis. Asterisks indicate *P* value ranges * *P* < 0.05, ** *P* <0.01, *** *P* < 0.001. Colour represents the type of genomic alteration. **e.** Mutual exclusivity and co-occurrence of truncal driver gene mutations and chromosome arm somatic copy number alterations in predominantly low/mid-grade tumours (n = 116). Colour of the edge represents the relationship (mutual exclusivity vs co-occurrence). The negative log of the *q* value (BH method) is represented in colour scale within each tile. Relationships with *q* < 0.1 are shown and asterisks indicate *q* value ranges * *q* < 0.05, ** *q* <0.01. **f.** Comparison of ploidy adjusted mean copy number of chromosomal arm and driver genes between high-grade and low/mid-grade predominant tumours. Fixed effect coefficients of the linear mixed effect model with each tumour as a random effect are displayed on the x-axis, and the negative log of the *q* value (BH method) is displayed on the y-axis. Colour represents the sign of the mean ploidy adjusted copy number, stratified with predominance of high-grade and low/mid-grade patterns. Data points with *q* value ≥ 0.05 are coloured in grey. Horizontal red dashed line represents *q* = 0.05. **g.** Gene set enrichment analysis of Hallmark gene sets between predominantly high- and low/mid-grade tumours. The normalised enrichment score is displayed on the x-axis and indicates the enrichment for a given gene set. Gene sets with *q* < 0.25 (BH method) are shown.

To elucidate the characteristics of each growth pattern in LUAD, the clinical, pathological, and genomic features across the different predominant subtypes at the whole tumour level were analysed (Fig. 1a, Extended Data Fig. 1l, Supplementary Table 1-3).

The proportion of high-grade patterns (solid, cribriform, and micropapillary) within a tumour, based on sectional area, was assessed in the context of variables associated with subclonal architecture and genomic instability (Fig. 1b, Methods). In TRACERx, an increasing proportion of high-grade patterns at the whole tumour level was associated with higher truncal, but not subclonal, TMB (truncal TMB, Spearman’s rho = 0.25, *q* value (FDR adjusted *P* value) = 0.0012, subclonal TMB, rho = 0.11, *q* = 0.12). Features of chromosomal complexity and instability, including the mean weighted genome instability index (wGII), mean fraction of the genome subject to loss of heterozygosity (FLOH) calculated across multiple regions within a tumour, and the percentage of subclonal SCNAs calculated as the fraction of the aberrant genome which is heterogeneous across tumour regions, were significantly associated with the proportion of high-grade patterns within a tumour (wGII, rho = 0.15, *q* = 0.039; FLOH, rho = 0.35, *q* = 2.9 × 10^-6^, % subclonal SCNA, rho = 0.32, *q* = 1.5 × 10^-5^, Methods) (Fig. 1b). Similar results were observed when using an orthogonal tool (Sequenza)^12^ for SCNA analysis (Extended Data Fig. 3a-c, Methods).

In our companion manuscripts, we show that metastasizing subclones tend to be spread across tumour regions, and a recent subclonal expansion score, defined as the largest cancer cell fraction (CCF) of any subclone terminal to the phylogenetic tree in any tumour region, is associated with shorter disease-free survival^10,11^. In LUAD, measures capturing the presence of a recent subclonal sweep and associated mutational homogeneity within individual tumour regions were significantly associated with an increasing proportion of high-grade patterns (Fig. 1b). These included the number of subclonal mutations which are clonal in at least one region (rho = 0.22, *q* = 0.0035) and the recent subclonal expansion score^10^ (rho = 0.16, *q* = 0.033, Methods). Conversely, the subclonal diversity index, a metric reflective of the number of coexisting subclones in a region and the absence of large recent clonal expansions, was lower in those tumours with a higher proportion of high-grade patterns (minimum subclonal diversity per tumour, rho = -0.23, *q* = 0.0030, Methods). Ki-67 fraction was also significantly associated with an increasing proportion of high-grade patterns (rho = 0.51, *q* = 1.6 × 10^-11^), consistent with previous reports^7,13^.

Taken together, these data suggest that while different regions from a high-grade tumour are frequently genomically distinct and can harbour distinct SCNAs, individual regions tend to be highly proliferative and clonally pure, potentially reflective of large intra-regional recent subclonal expansions.

When the proportion of each individual growth pattern was compared against these genomic features, the presence of highly proliferative and recent subclonal expansion was most strongly associated with the proportion of the solid-pattern component within a tumour (Ki-67 fraction, rho = 0.51, *q* = 8.1 × 10^-11^, recent subclonal expansion score, rho = 0.19, *q* = 0.026) (Fig. 1c). Intriguingly, although micropapillary pattern is regarded as a high-grade pattern that is associated with poor prognosis^1,5^, an increasing proportion of micropapillary-pattern component was associated with increasing subclonal diversity and a lack of evidence for clear subclonal expansions (minimum subclonal diversity per tumour, rho = 0.22, *q* = 0.0091, recent subclonal expansion score, rho = -0.18, *q* = 0.028), suggesting distinct biology and clonal evolutionary characteristics between high-grade solid and micropapillary growth patterns.

### Determinants of inter-tumoural growth pattern heterogeneity

**Extended Data Fig. 2 |:**
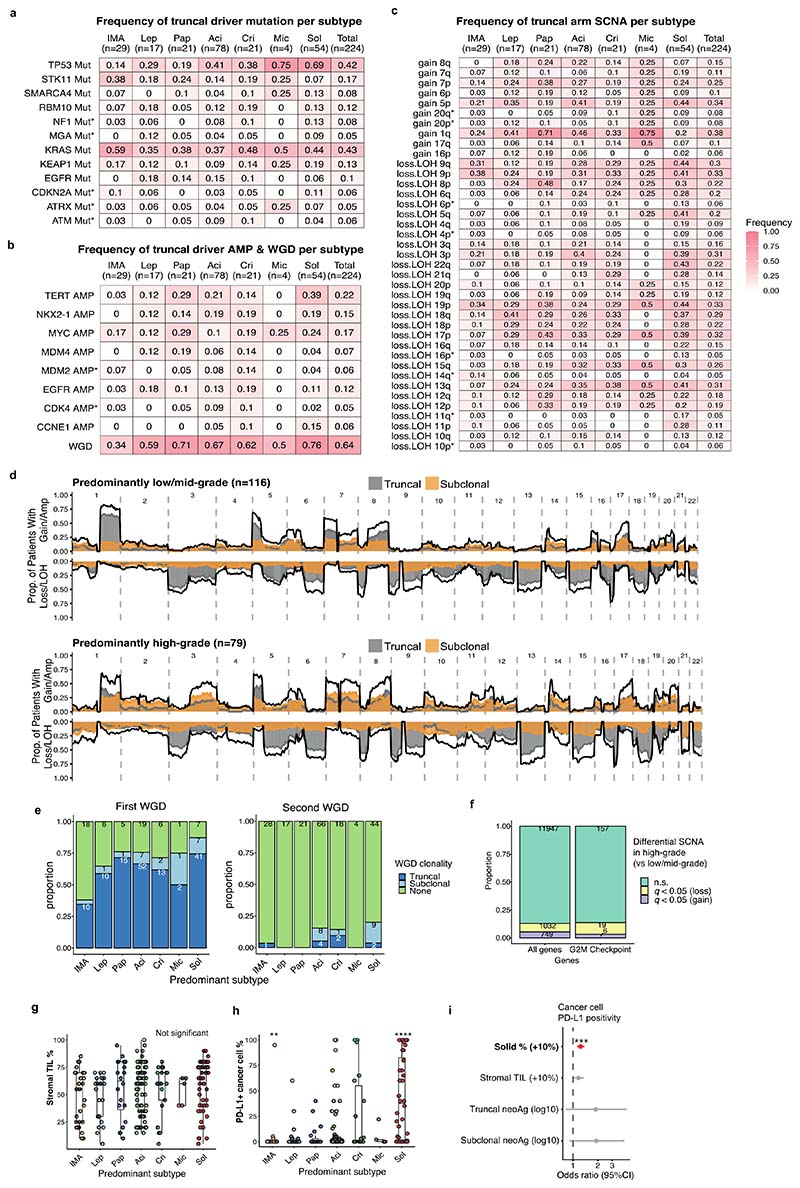
Genomic correlates of LUAD predominant subtypes **a-c.** Frequency of truncal driver mutations **(a)**, truncal driver gene somatic copy number alterations (SCNAs) (AMP, amplification) and whole genome doubling (WGD) **(b),** and chromosomal arm level SCNAs (gain/loss&LOH) **(c)** in LUAD predominant subtypes. Recurrent truncal alterations observed in more than 5% of the tumours in the cohort are shown. Asterisks represent the alterations observed in fewer than 10 tumours in both predominantly high- and low/mid-grade predominant tumours. Colour scale represents the frequency of the alteration observed within each subtype. **d**. Across-genome plots showing the frequency of truncal and subclonal SCNAs of low/mid-grade predominant tumours (top) and high-grade predominant tumours (bottom). Within each tumour type, the proportion of patients with gains or amplifications (top) and loss/LOH events (bottom) for each chromosome are described. The black line indicates the total (namely the sum of truncal and subclonal) proportion of tumours with SCNAs; the yellow and grey lines or shades indicate the proportion of tumours with subclonal and truncal gains, respectively. **e.** The frequency of first and second WGD across LUAD predominant subtypes. **f.** Number of genes with differential SCNA between high-grade and low/mid-grade predominant tumours. G2M checkpoint-related genes were not differentially gained in predominantly high-grade tumours (*P* = 0.20, chi-square goodness of fit test). **g,h.** Comparison of stromal TIL scores **(g)** and PD-L1 expression on cancer cells measured by IHC staining **(h)** across LUAD predominant subtypes. Each predominant subtype was compared against all other subtype tumours. Centre line, median; box limits, upper and lower quartiles; whiskers, 1.5× interquartile range. *P* values were corrected for multiple testing according to Benjamini-Hochberg and asterisks indicate *q* value ranges * *q* < 0.05, ** *q* <0.01, *** *q* < 0.001, **** *q* < 0.0001. **i.** Adjusted odds ratios for cancer cell PD-L1 positivity (≥ 1%) estimated by multivariable logistic regression model. Asterisks indicate type II ANOVA *P* value ranges * *P* < 0.05, ** *P* <0.01, *** *P* < 0.001. Statistically significant covariates are indicated in bold.

**Extended Data Fig. 3 |:**
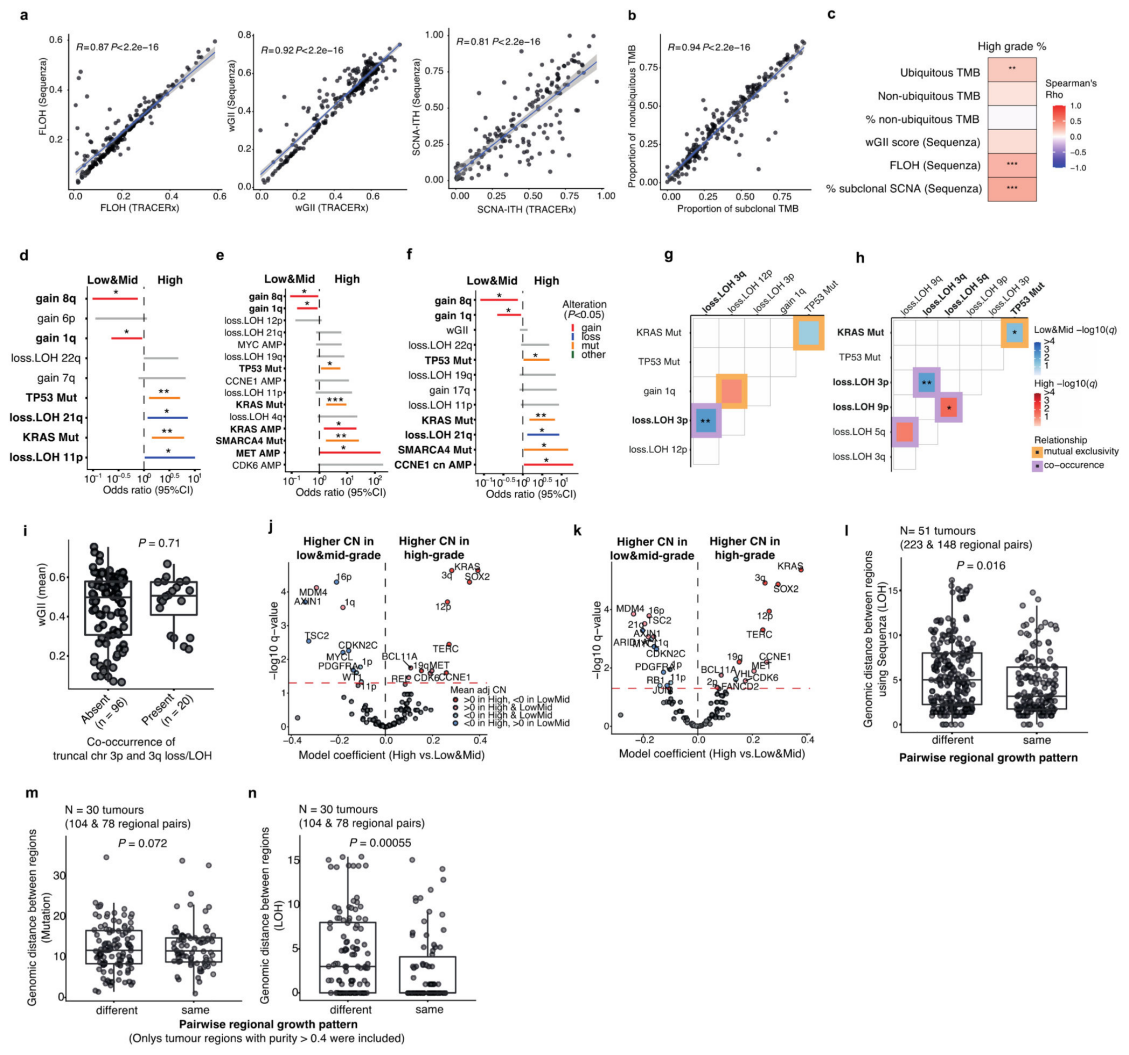
Validation of SCNA analysis using orthogonal methods **a-b.** Comparison of ITH metrics calculated using TRACERx analytical pipeline vs orthogonal methods. (**a**) Comparison of fraction of the genome subject to loss of heterozygosity (FLOH), weighted genome instability index (wGII), and somatic copy number aleration intra-tumour heterogeneity (SCNA-ITH) by SCNA profiles generated by the TRACERx pipeline (based on ASCAT^46^ with additional multi-sample SCNA estimation approach^10,47^) against SCNA profiles generated by Sequenza^12^ and (**b**) a comparison of % subclonal tumour mutational burden (TMB) using the TRACERx pipeline (clonality inferred by the modified version of PyClone^48^) vs % non-ubiquitous TMB. **c**. Correlation of genomic variables calculated using orthogonal methods and the proportion of high-grade patterns within each tumour. Colour scale reflects Spearman’s rank correlation coefficient (rho). Correlation *P* values were corrected for multiple testing according to Benjamini-Hochberg (BH) and asterisks indicate *q* value ranges * *q* < 0.05, ** *q* <0.01, *** *q* < 0.001, **** *q* < 0.0001. **d-f**. Adjusted odds ratios of truncal genomic alterations associated with the predominance of high-grade patterns. Genomic alterations selected by the model simplification are shown when (**d**) truncal alterations observed in more than 10% of the tumours in the cohort are included in the analysis, or when (**e**) SCNA profiles generated by Sequenza^12^ are used, or when (**f**) wGII is added to the model shown in Fig. 1d. Asterisks indicate type II ANOVA *P* value ranges * *P* < 0.05, ** *P* <0.01, *** *P* < 0.001. Colour represents the type of genomic alteration. Statistically significant alterations are indicated in bold. **g-h**. Mutual exclusivity and cooccurrence of truncal driver gene alterations and chromosome arm somatic copy number alterations when (**g**) truncal alterations observed in more than 10% of the tumours in the cohort are included in the analysis, or when (**h**) SCNA profiles generated by Sequenza^12^ are used. Colour of the edge represents the relationship (mutual exclusivity vs co-occurrence) and the negative log of the *q* value (BH method) is represented in blue colour scale in predominantly low/mid-grade tumours and red colour scale in predominantly high-grade tumours. Relationships with *q* < 0.1 are shown and asterisks indicate *q* value ranges * *q* < 0.05, ** *q* <0.01. Covariates in statistically significant relationships are indicated in bold. **i**. Comparison of wGII between tumours with and without co-occurrence of truncal loss/LOH of chromosome 3p and 3q in predominantly low/mid-grade tumours. *P* value was calculated using Wilcoxon rank sum test. **j-k**. Comparison of ploidy adjusted mean copy number of chromosomal arm and driver genes between high-grade and low/mid-grade predominant tumours, (**j**) using SCNA profiles generated by Sequenza^12^, and (**k**) adding wGII to the regression model. Fixed effect coefficients of the linear mixed effect model with each tumour as a random effect are displayed on the x-axis, and the negative log of the *q* value (BH method) is displayed on the y-axis. Colour represents the sign or the mean ploidy adjusted copy number, stratified with high-grade and low/mid-grade predominance. Data points with *q* value ≥ 0.05 are coloured in grey. Horizontal red dashed line represents *q* = 0.05. **l-n**. Genomic distance between regions calculated by LOH detected by Sequenza (**l**, n = 51 tumours) and genomic distance calculated by mutation (**m**) and LOH (**n**) only including tumour regions with purity > 0.4 (n = 30 tumours). Each point represents a distance between a pair of regions in a tumour. Tumours with regions containing both different subtype pair(s) and same subtype pair(s) are included in the analysis. Centre line, median; box limits, upper and lower quartiles; whiskers, 1.5× interquartile range. P values were calculated using a linear mixed effects model, with each tumour as a random effect.

To explore the evolutionary determinants of growth patterns in LUAD, the presence of truncal genomic alterations was correlated with the predominant pattern within a tumour, assuming that specific early genomic events may influence the subsequent growth pattern. In total, 13 truncal driver alterations (7 mutations, 6 amplifications) and 31 chromosomal arm-level truncal SCNAs (8 gains and 23 losses/LOHs) observed in at least 5% of the cohort and at least 10 tumours in either predominantly high-or low/mid-grade tumours were included in a logistic regression analysis (Extended Data Fig. 2a-c, Methods). Previous studies have reported an increased frequency of *TP53*^6,14,15^ and KRAS^16^ mutations in solid predominant tumours. In the TRACERx cohort, in addition to truncal *TP53* and *KRAS* driver mutations, truncal *SMARCA4* mutation, truncal *CCNE1* amplification, and truncal loss/LOH of chromosome 21q were associated with predominantly high-grade pattern tumours, while truncal gains of 1q and 8q were associated with predominantly low/mid-grade tumours (Fig. 1d). Similar results were observed when only including truncal alterations observed in at least 10% of the cohort, applying an orthogonal tool (Sequenza)^12^ for SCNA profiling, or after adding genomic instability as a covariate in the regression model (Extended Data Fig. 3d-f, Methods).

Co-occurrence of truncal loss/LOH of 3p and 3q was observed in predominantly low/mid-grade tumours but not in predominantly high-grade tumours, suggesting the whole loss of chromosome 3 may be an early evolutionary event specifically in predominantly low/mid-grade tumours (Fig. 1e, Extended Data Fig. 3g-h, Methods). Co-occurrence of truncal 3p and 3q loss/LOH within predominantly low/mid-grade tumours was not associated with higher wGII, suggesting that co-occurrence of the loss of these chromosome arms is not a reflection of genomic instability (*P* = 0.71, Wilcoxon rank sum test) (Extended Data Fig. 3i). These results may indicate that a co-occurrence of 3p and 3q losses, possibly reflecting the loss of one allele of chromosome 3 as one event, is a distinct evolutionary route to predominantly low/mid-grade tumours.

To capture SCNAs with <5% frequency in the cohort, which would not have been included in the regression analysis, an expanded analysis of the driver alterations and chromosomal arm-level copy number alterations varying between predominantly high- and low/mid-grade tumours was carried out (Methods). The characteristics most strongly associated with predominantly high-grade tumours versus predominantly low/mid-grade tumours were gains of chromosome arms 3q (whole arm 3q, encompassing the *SOX2* and *TERC* genes) and 12p (whole arm 12p, encompassing the *KRAS* gene, consistent with a previous report^6^), both of which were often seen as loss/LOH in predominantly low/mid-grade tumours (Fig. 1f, Extended Data Fig. 2c-d). Similar results were observed when applying an orthogonal tool (Sequenza)^12^ for SCNA profiling, or after adding genomic instability as a covariate in the regression model (Extended Data Fig. 3j-k, Methods).

In 59% of lepidic predominant invasive adenocarcinomas, a truncal whole genome doubling (WGD) event was detected (Extended Data Fig. 2b-e). By contrast, in the preinvasive lesions, atypical adenomatous hyperplasia (AAH) and adenocarcinoma in situ (AIS), analysed in the TRACERx pre-invasive cohort, <10% demonstrated evidence of WGD^17^. These data highlight the potential biological differences between alveolar wall surface growth (lepidic pattern) in the setting of pre-invasive disease versus a partly invasive-pattern adenocarcinoma. This suggests that WGD may occur prior to malignant transition from pre-invasive to invasive disease, as previously shown in oesophageal adenocarcinoma^18^.

At the transcriptome level, consistent with our observation that increasing Ki-67 fraction is associated with the proportion of high-grade patterns observed at the whole tumour level (Fig. 1b), gene-set enrichment analyses revealed the greatest differential expression between high-grade and low/mid-grade predominant tumours involved genes related to cell cycle and cell proliferation (Fig. 1g, Methods), including upregulated G2M checkpoint, mTORC1 signalling and PI3K-AKT-mTOR signalling. Notably, predominantly high-grade tumours did not necessarily harbour increased copy number of cell cycle genes compared with predominantly low/mid-grade tumours (significantly increased copy number in high-grade predominant tumours compared with low/mid-grade predominant tumours, G2M checkpoint genes, 6/182; all genes, 749/13728; *P* = 0.20, chi-square goodness of fit test) (Extended Data Fig. 2f, Methods), suggesting that the overexpression of cell cycle genes in predominantly high-grade tumours may not be directly driven by gains of cell cycle genes.

Although there was no association observed between stromal TIL infiltration and the predominant growth pattern (Extended Data Fig. 2g), cancer cell PD-L1 expression, assessed by immunohistochemistry, was significantly higher in solid predominant tumours than all other histological subtypes (*q* = 4.8 × 10^-7^, Wilcoxon rank sum test, Extended Data Fig. 2h), as previously reported^14^. The proportion of solid pattern remained significantly associated with cancer cell expression of PD-L1 after adjustment for potential confounders, including stromal TILs and neoantigen burden (OR=1.23 [95%CI 1.11-1.38] per 10% increase, *P* = 3.3 × 10^-5^, ANOVA, Methods) (Extended Data Fig. 2i), suggesting that overexpression of PD-L1 in solid predominant tumours may be driven by cancer cell intrinsic characteristics, such as AKT-mTOR pathway activation^19^.

### Morphological intra-tumour heterogeneity reflects genomic intra-tumour heterogeneity

The data presented thus far have focussed on growth pattern characterisation at the whole tumour level. However, the multi-region sampling and sequencing in TRACERx allows for the analysis of intra-tumour growth pattern heterogeneity in the context of the genomic and transcriptomic landscape. A total of 200 tumours had regional histology data available, amounting to 603 pathological regions used in this analysis (Fig. 1a, Extended Data Fig. 1j, Supplementary Table 4).

**Fig. 2 |:**
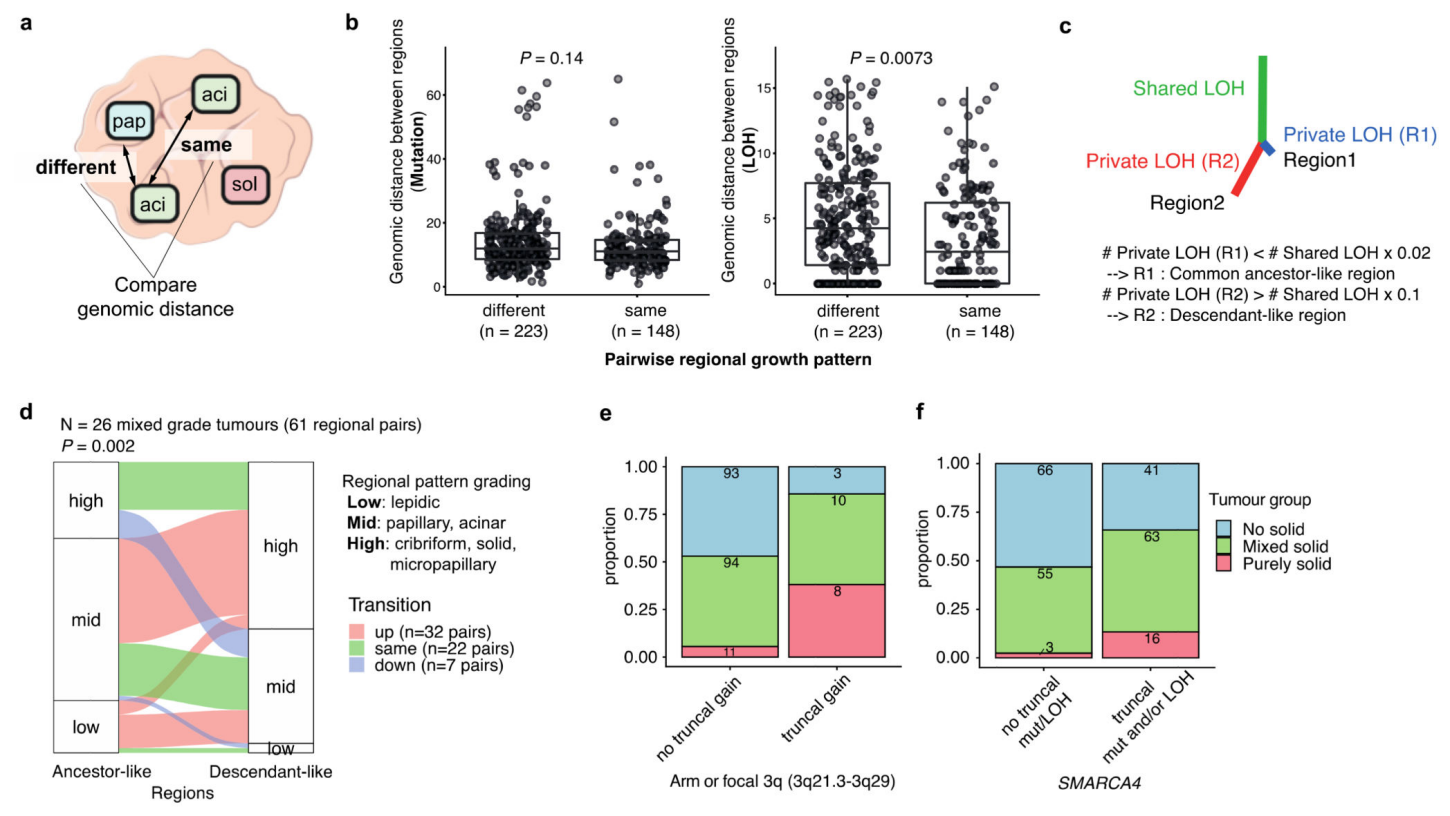
Morphological intra-tumoural heterogeneity reflects genomic heterogeneity **a.** Schematic illustrating regions with different or the same growth patterns within each tumour. **b.** Genomic distance between regions calculated by presence of somatic mutation (**left**, n = 51 tumours) and LOH (**right**, n = 51 tumours). Genomic distances between identical (same) growth pattern regions and different growth pattern regions were compared. Each point represents a distance between a pair of regions in a tumour, and the number of regional pairs is shown under each group. Tumours with regions containing both different growth pattern pair(s) and same growth pattern pair(s) are included in the analysis. Centre line, median; box limits, upper and lower quartiles; whiskers, 1.5× interquartile range. *P* values were calculated using a linear mixed effects model, with each tumour as a random effect. **c.** Schematic illustrating inference of ancestor-like and descendant-like regional pairs using shared and private LOH profiles per cytoband. After building a LOH tree, if a branch length of Region1 (R1) is shorter than 2% of the trunk length, namely if R1 has private LOH burden less than 2% of the shared LOH burden, then R1 is inferred as a common ancestor-like region. Conversely, if a branch length of Region2 (R2) is longer than 10% of trunk length, namely if R2 has private LOH burden more than 10% of shared LOH burden, then R2 is inferred as a descendant-like region. **d.** Comparison of growth pattern (grade) between inferred ancestral-like and descendant-like regions. Only tumours with mixed pattern grades are included in the analysis. Colour represents the transition of grade from ancestor-like to descendant-like region. Empirical *P* value was calculated using a permutation test (1000 permutations, randomising growth patterns within each tumour, Monte-Carlo procedure). **e-f.** Proportion of tumours which are purely solid, mixed pattern with solid component, and without any solid component, compared between the tumours with and without (**e**) truncal gain of arm or focal 3q (3q21.3-3q29) and (**f**) truncal *SMARCA4* mutation and/or LOH.

To determine whether the variation in morphology within a tumour reflected the degree of subclonal alterations, genomic distances based on mutations (SNVs and indels) and LOH were calculated between regions of the same tumour and explored in relation to the different regional growth patterns (Fig. 2a, Methods). Consistent with findings from the whole tumour analysis that FLOH, but not subclonal TMB, was associated with the proportion of high-grade patterns, the genomic distance between regions with different growth patterns was significantly greater than the genomic distance between regions with the same growth pattern when calculated using LOH (*P* = 0.0073, linear mixed effect model, ANOVA), but not when using mutations (*P* = 0.14) (Fig.2b, Extended Data Fig. 3ln). This may reflect the fact that LOH is irreversible. By contrast, truncal mutations may be subject to copy number loss, rendering them subclonal and potentially less reliable as a marker of evolutionary divergence.

**Extended Data Fig. 4 |:**
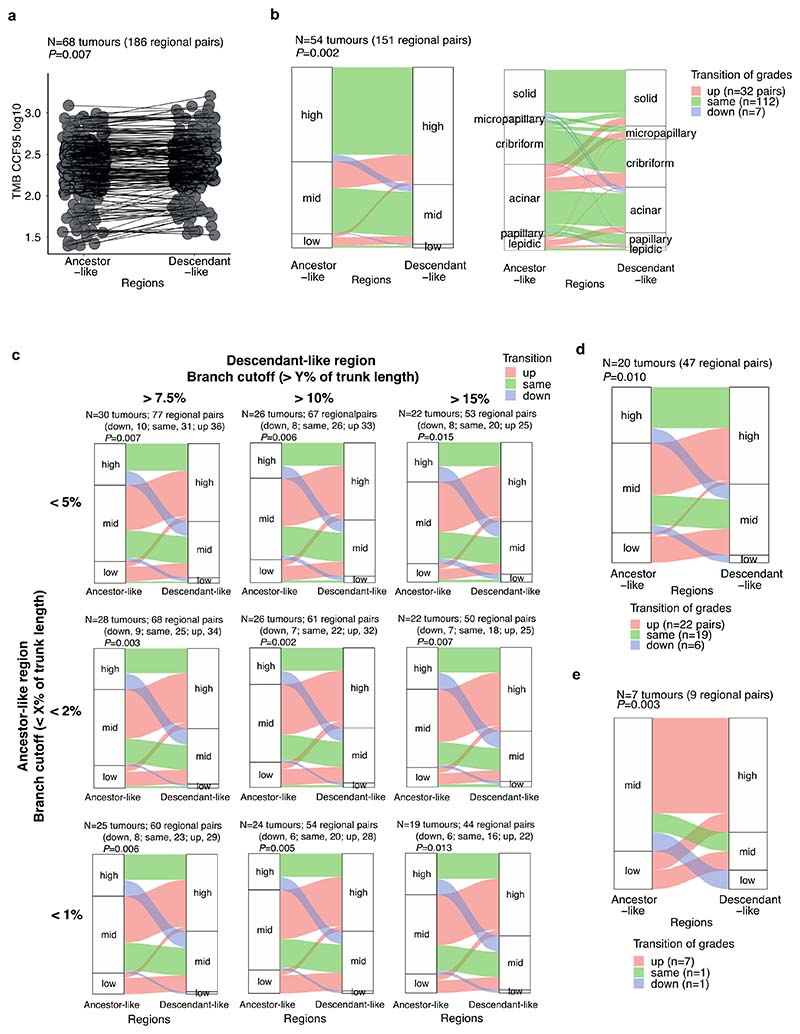
Inference of ancestor-like and descendant-like regional pairs in the primary tumour **a**. Comparison of tumour mutational burden (TMB) between ancestor-like and descendant-like regions. Each line represents an ancestor-descendant-like regional pair. Each point represents one region and the plotted points were duplicated for regions associated with multiple ancestor-descendant-like pairs within a tumour. To assess the mutational burden shared in the majority of the cancer cells in the region, mutations with estimated cancer cell fraction ≥ 95% were counted (TMB CCF95). Enrichment of higher TMB in descendant-like regions compared with the paired ancestor-like regions was evaluated by permutation test (1000 permutations, randomising TMB within each tumour, Monte-Carlo procedure). **b**. Comparison of growth pattern by grades (**left**) and by the six growth patterns (**right**) between inferred ancestral-like and descendant-like regions. Tumours with single grades are included in the analysis. Colour represents the transition of grade from ancestral-like to descendant-like region. Empirical *P* value was calculated using a permutation test (1000 permutations, randomising growth patterns within each tumour, Monte-Carlo procedure). **c**. Comparison of regional growth pattern grade in ancestor-descendant-like pairs, inferred by various cutoffs of private LOH branch length proportional to the trunk (shared LOH). All combinations of cutoff for ancestor-like and descendant-like inference shown in the figure yielded empirical *P* value < 0.05 (1000 permutations, Monte-Carlo procedure) when the enrichment of lower-to-higher grade transition (upward transition) was tested. P values were not adjusted for the multiple comparisons shown in this panel. **d**. Comparison of regional pattern grade in ancestor-descendant-like pairs, inferred by LOH profile generated by Sequenza ^12^. **e**. Comparison of regional pattern grade in ancestor-descendant-like pairs, inferred by both LOH profile and mutational profile (CCF ≥ 95%).

To assess whether different morphological patterns reflect an evolutionary trajectory from low-to high-grade pattern, we utilised the irreversible nature of LOH to determine ancestor-descendant-like relationships in tumour regions and the context of their respective morphological grades (Fig. 2c, Methods). This was based on the assumption that, although a tumour region is not directly evolved from another region, in some regional pairs one region will harbour a common ancestral-like clone while the other region may harbour a descendant-like clone of the common ancestor. We identified 151 regional pairs with ancestor-descendant-like relationships within 54 tumours using LOH profiles. Comparison of the mutational profiles of these ancestor-descendant-like pairs revealed that descendant-like regions harboured additional mutations at a high cancer cell fraction (CCF ≥ 95%) compared to their ancestor-like counterpart regions (*P* = 0.007, permutation test, Methods) (Extended Data Fig. 4a). Within the mixed pattern grade tumours, descendant-like regions exhibited a significantly higher grade compared with their respective ancestral-like regions (*P* = 0.002, permutation test, Methods) (Fig. 2d, Extended Data Fig. 4b). This association remained significant when various cut-offs were applied to infer ancestor-descendant-like regional pairs (Extended Data Fig. 4c), or when using an orthogonal tool (Sequenza)^12^ for LOH profiling (Extended Data Fig. 4d). This association also remained significant when ancestor-descendant-like regional pairs were inferred using a combination of LOH and mutational profiles (Extended Data Fig. 4e, Methods). These data suggest that heterogeneity in morphological patterns may be partially underpinned by genomic alterations. Notably, LUADs did not always follow an evolutionary route towards higher grade patterns, suggesting that, although very rare (7/151 ancestor-descendant-like regional pairs) (Extended Data Fig. 4b), tumours may transition to lower grade patterns during subclonal evolution.

### Distinct evolutionary trajectory of purely solid tumours

Tumours lacking any adenocarcinoma architectural differentiation, namely purely solid pattern tumours, are typically classified as adenocarcinoma on the basis of immunohistochemical expression of TTF-1 alone. Molecular characteristics of purely solid tumours are poorly understood, and the evolutionary trajectory of purely solid tumours, especially whether these have evolved from mixed pattern adenocarcinomas which include a solid component, remains unclear. To address this, we explored if solid-pattern regions in purely solid tumours and solid-pattern regions in mixed pattern tumours harboured similar genomic and transcriptomic features, and if purely solid tumours were genomically distinct from mixed pattern tumours with a solid component.

**Extended Data Fig. 5 |:**
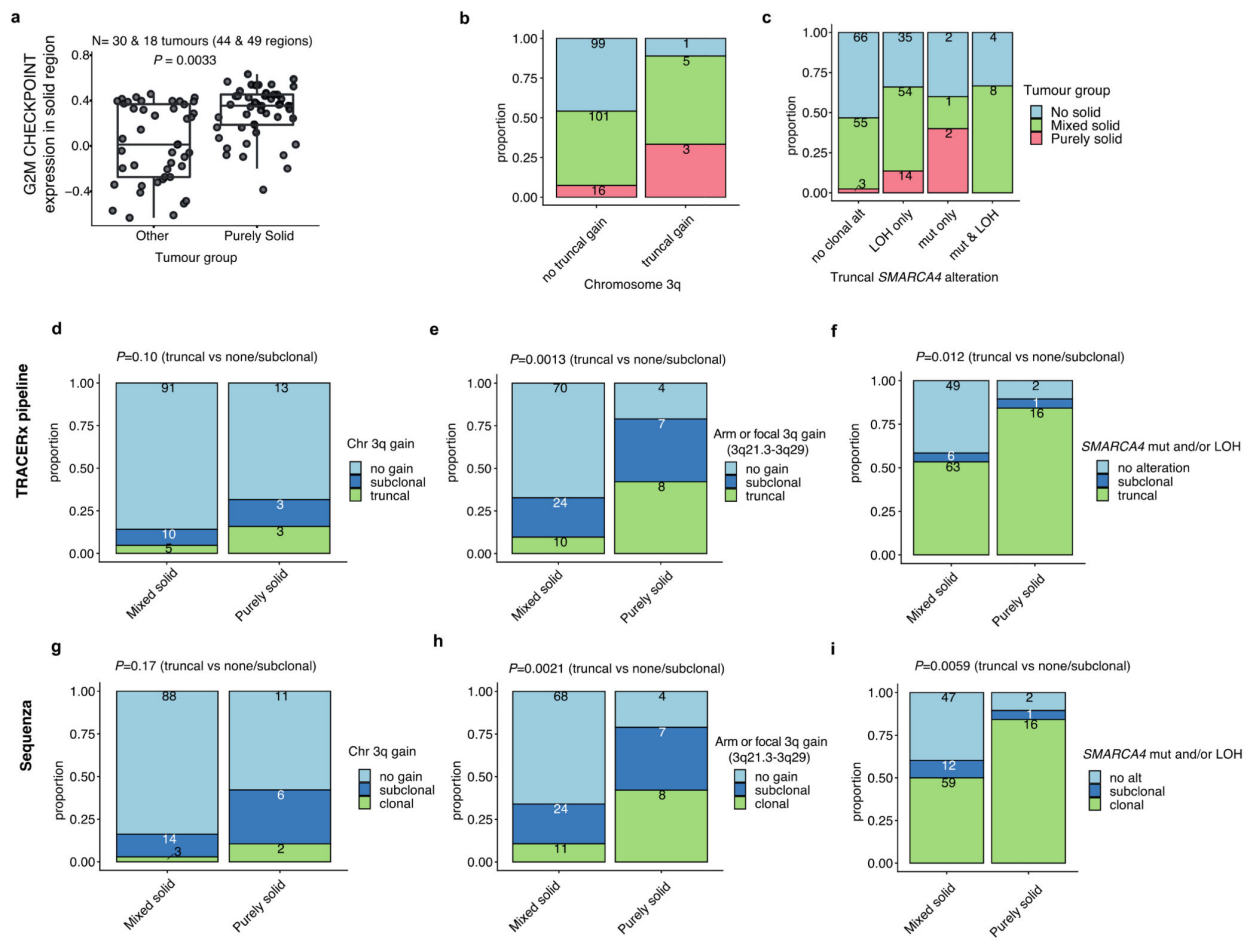
Characterisation of purely (homogenously) solid tumours **a.** Comparison of G2M checkpoint gene expression in solid-pattern regions within purely solid tumours and mixed pattern tumours as defined by both diagnostic and regional growth pattern assessment. Centre line, median; box limits, upper and lower quartiles; whiskers, 1.5× interquartile range. *P* values were calculated using a linear mixed effects model, with each tumour as a random effect. **b-c.** Proportion of tumours which are purely solid, mixed pattern with solid component, and without any solid component, compared (**b**) between tumours with and without truncal gain of chromosome arm 3q and (**c**) across the tumours stratified by truncal *SMARCA4* mutation and/or loss of heterozygosity (LOH). **d-e.** Comparison of the frequency of truncal copy number gain of (**d**) chromosome arm 3q and (**e**) arm or focal 3q (3q21.3-3q29) between mixed pattern tumours with solid component and purely solid tumours. **f**. Comparison of the frequency of truncal *SMARCA4* mutation and LOH between mixed pattern tumours with solid component and purely solid tumours. **g-h.** Comparison of the frequency of copy number gain of (**g**) chromosome arm 3q and (**h**) arm or focal 3q (3q21.3-3q29) between mixed pattern tumours with solid component and purely solid tumours using somatic copy number alteration (SCNA) profiles generated by Sequenza. **i**. Comparison of the frequency of *SMARCA4* mutation and LOH between mixed pattern tumours with solid component and purely solid tumours using SCNA profiles generated by Sequenza.

Solid regions in purely solid tumours showed overexpression of G2M checkpoint genes compared with solid regions in other mixed growth pattern tumours (*P* = 0.0033, linear mixed effect model, ANOVA) (Extended Data Fig. 5a). At the whole tumour level, the presence of truncal 3q gain (purely solid vs other tumours, OR = 6.2 [95%CI 0.91-32.3], *P* = 0.031, Fisher’s exact test), especially the truncal focal gain of 3q21.3-3q29 which GISTIC2.0 analysis^20^ detected as a significant peak in tumour regions with solid pattern (purely solid vs other tumours, OR = 10.2 [95%CI 3.0-34.1], *P* = 7.1 × 10^-5^, Fisher’s exact test, Methods), and truncal *SMARCA4* mutations and/or LOH (purely solid vs other tumours, OR = 6.2 [95%CI 1.7-33.9], *P* = 0.0015, Fisher’s exact test) were associated with increased likelihood of purely solid tumours (Fig. 2e-f, Extended Data Fig. 5b-i). These results suggest that purely solid tumours have genomic alterations and an evolutionary trajectory distinct from mixed growth pattern tumours with a solid component.

### Evolution of LUAD growth patterns from primary tumour to metastasis

**Fig. 3 |:**
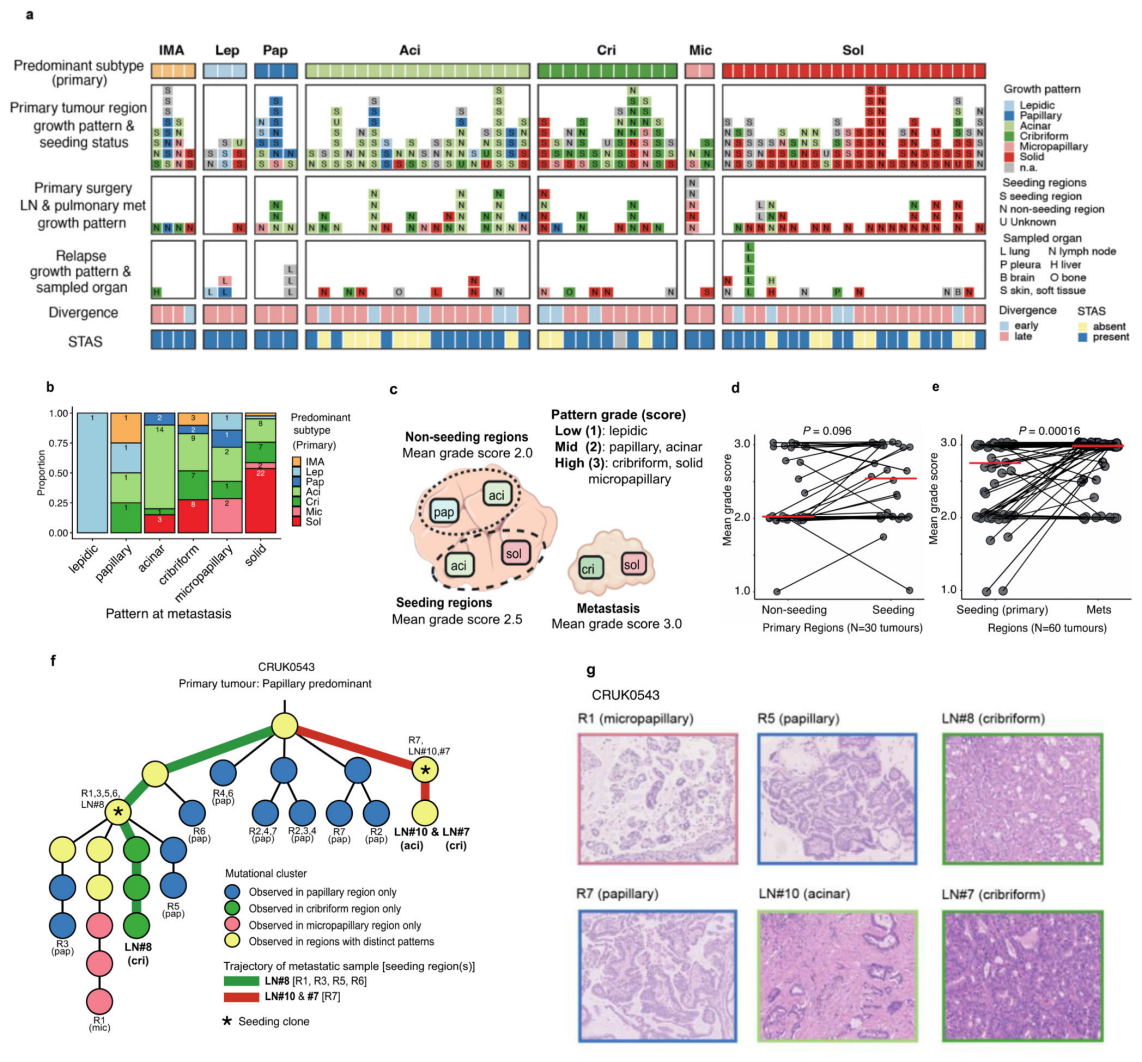
Evolution of LUAD growth patterns through metastasis **a.** Overview of metastasis samples in the TRACERx LUAD cohort (n = 65 tumours). Growth pattern and the presence of seeding clones in primary tumour, growth pattern and the site of metastasis samples, timing of divergence of the metastasising clone, and presence of the tumour spread through air space (STAS) in the primary tumour are shown. **b.** Frequency of metastasis samples analysed according to the growth pattern at metastasis. The y-axis represents the proportion of the metastatic samples, with the colour representing the predominant subtype of the primary tumour. Multiple metastasis samples from the same primary tumour are counted independently. **c.** Schematic showing the calculation of mean grade scores of non-seeding regions and seeding regions in the primary tumour, as well as metastatic samples. Grade scores of 1, 2, and 3 were given for low-, mid- and high-grade patterns respectively, and mean scores per group were calculated for each tumour. **d.** Comparison of growth patterns between seeding and non-seeding regions in primary tumours. Growth patterns were transformed into scores (1: low-grade, 2 : mid-grade and 3: high-grade) and mean scores of non-seeding region(s) and seeding region(s) were calculated for each tumour, as described in Fig. 3c. Tumours harbouring at least one seeding and non-seeding region with growth pattern annotation were included in the analysis (n = 30). Mean scores of growth patterns in seeding and non-seeding regions were calculated. The median is indicated by the red horizontal line. A two-sided Wilcoxon signed-rank test was used. **e.** Comparison of growth pattern between metastasis and the primary tumour seeding regions (n = 60). The median is indicated by the red horizontal line. A two-sided Wilcoxon signed-rank test was used. **f.** Example of phylogenetic tree (CRUK0543) including multiple metastases to lymph nodes resected at surgery. Each node in the tree represents a mutational cluster and their colour indicates the following: blue, mutational cluster only seen in papillary region (primary tumour regions R2, 3, 4, 5, 6, 7); pink, mutational clusters only seen in micropapillary region (primary tumour region R1); green, mutational clusters only seen in cribriform regions (metastatic LN #8); yellow, mutational clusters seen in regions with different patterns. LN#10 (acinar) and LN#7 (cribriform) were predicted to have identical mutational clones. Asterisks represent most recent common ancestors of primary tumour regions and metastases (seeding clones). Terminal clusters of each branch and seeding clones are annotated with the region name where the cluster is harboured and with the growth pattern of the region in the brackets. **g**. Representative haematoxylin and eosin staining images from CRUK0543. R1, primary region with micropapillary pattern; R5 and R7, primary regions with papillary pattern; LN#10, metastatic lymph node with acinar pattern; LN#7 and #8, metastatic lymph nodes with cribriform pattern.

To determine the relationship between growth patterns in available matched primary and metastatic tumours, 121 metastatic samples from 65 patients were studied specifically with reference to the phylogeny of metastatic clones (Fig. 3a). Metastatic tumours consisted of lymph nodes (LNs) (n=83) and intrapulmonary metastasis (n=2) removed at the time of primary surgery, and sites of disease relapse sampled using diagnostic biopsies including surgical resection (n=36), and were subjected to centralised pathological review and subtyping (proportion of the samples pathologically reviewed for growth pattern, 107/121, 88%). The majority of metastatic samples displayed high-grade patterns (82/107, 77%) (Fig. 3a-b). Each primary tumour region was classified into metastasis seeding or metastasis non-seeding regions based on combined phylogenetic analysis^11^. In brief, seeding regions were defined as the primary tumour regions harbouring metastasis seeding clones, and the seeding clones were defined as the most recent shared clone between the primary tumour and metastasis.

Notably, in certain tumours multiple seeding clones were identified, and in certain cases these were spread across all tumour regions. Using a numerical score assigned to high, mid, and low-grade patterns, the mean grade for each seeding region was calculated and compared with the mean grade for each matched metastatic tumour region and primary tumour non-seeding regions (Fig. 3c). While no significant difference was observed using this numerical score to compare seeding and non-seeding regions in the primary tumour (*P* = 0.096, Wilcoxon signed-rank test) (Fig. 3d), the metastatic regions were typically the same or higher grade compared with their matched seeding regions (*P* = 1.6 × 10^-4^, Wilcoxon signed-rank test) (Fig. 3e). One such example was a papillary predominant primary adenocarcinoma (CRUK0543, Fig. 3f-g) in which two out of three metastatic lymph nodes (LN#7 and LN#8), deriving from different phylogenetic branches, showed high-grade (cribriform) pattern, while the majority (4/5) of the seeding regions showed mid-grade (papillary) pattern, suggestive of parallel evolution of growth patterns during the metastatic cascade.

**Extended Data Fig. 6 |:**
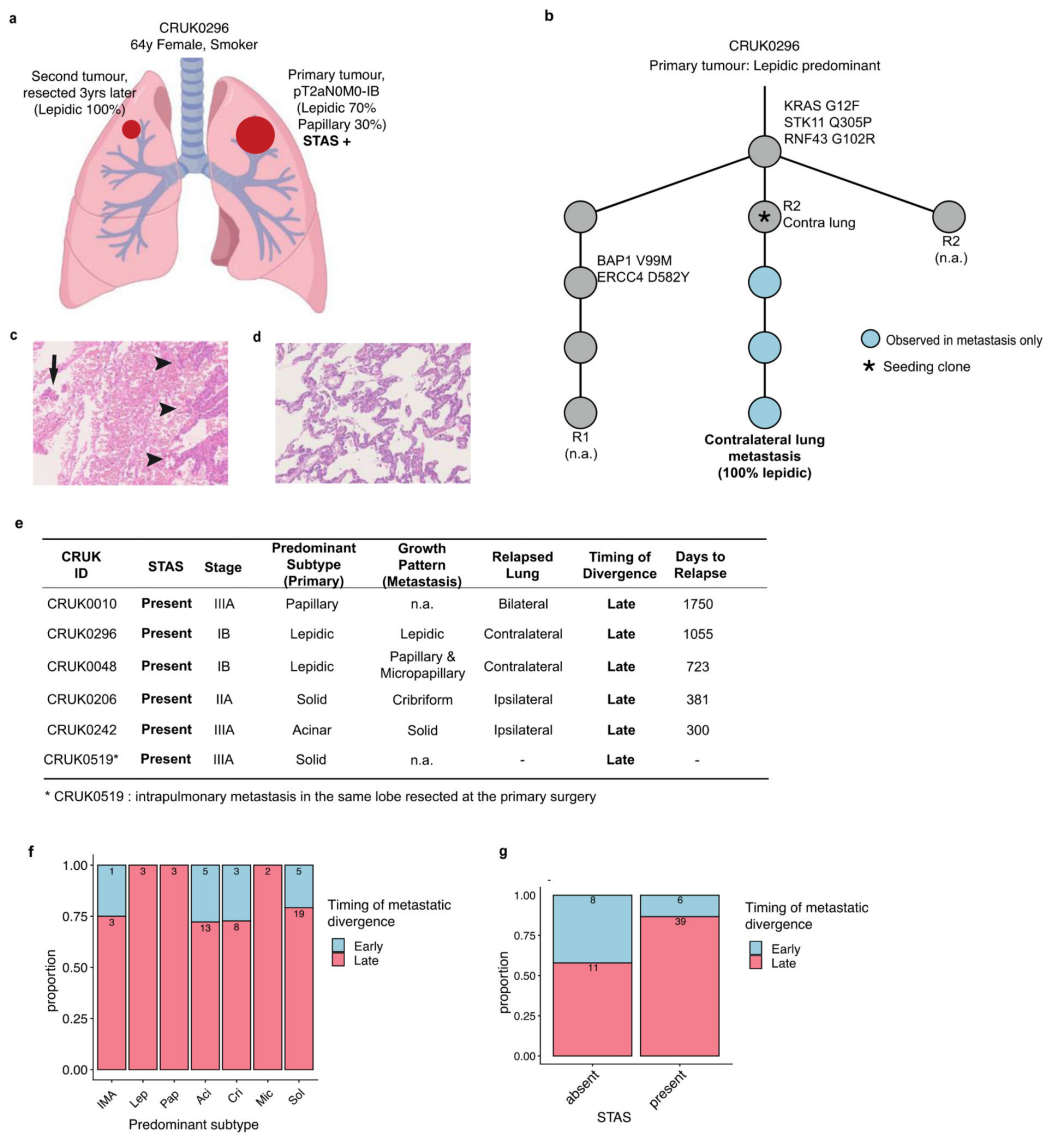
Analysis of morphology and genomics in metastasis samples **a**. Schematic of primary and secondary lung tumours in CRUK0296. Phylogenetic analysis confirmed the contralateral lung lesion to be a metastasis from the resected primary tumour three years ago. Tumour spread through air spaces (STAS) was positive in the primary tumour. **b.** Phylogenetic tree of a case having lung metastasis with pure lepidic appearance (CRUK0296). Driver mutations are shown in the figure at the concordant mutational cluster. Regional growth pattern is indicated in brackets; n.a., not available. **c.** Representative haematoxylin and eosin (H&E) slide of a primary tumour of CRUK0296 showing tumour border (arrowheads) and STAS (arrow). **d.** Representative H&E slide of metastasis tumour in the contralateral lung of CRUK0296, which showed a pure lepidic pattern. **e.** Characteristics of five patients having lung recurrence samples sequenced and one patient having an intrapulmonary metastasis resected and sequenced at the time of primary surgery. All six patients showed positive STAS in the primary tumours and phylogenetic analysis revealed late metastatic divergence. **f.** Proportion of the timing of seeding clone divergence across predominant subtypes of primary tumours. **g.** Frequency of late or early divergence of the metastatic clone compared between tumours with and without STAS.

**Extended Data Fig. 7 |:**
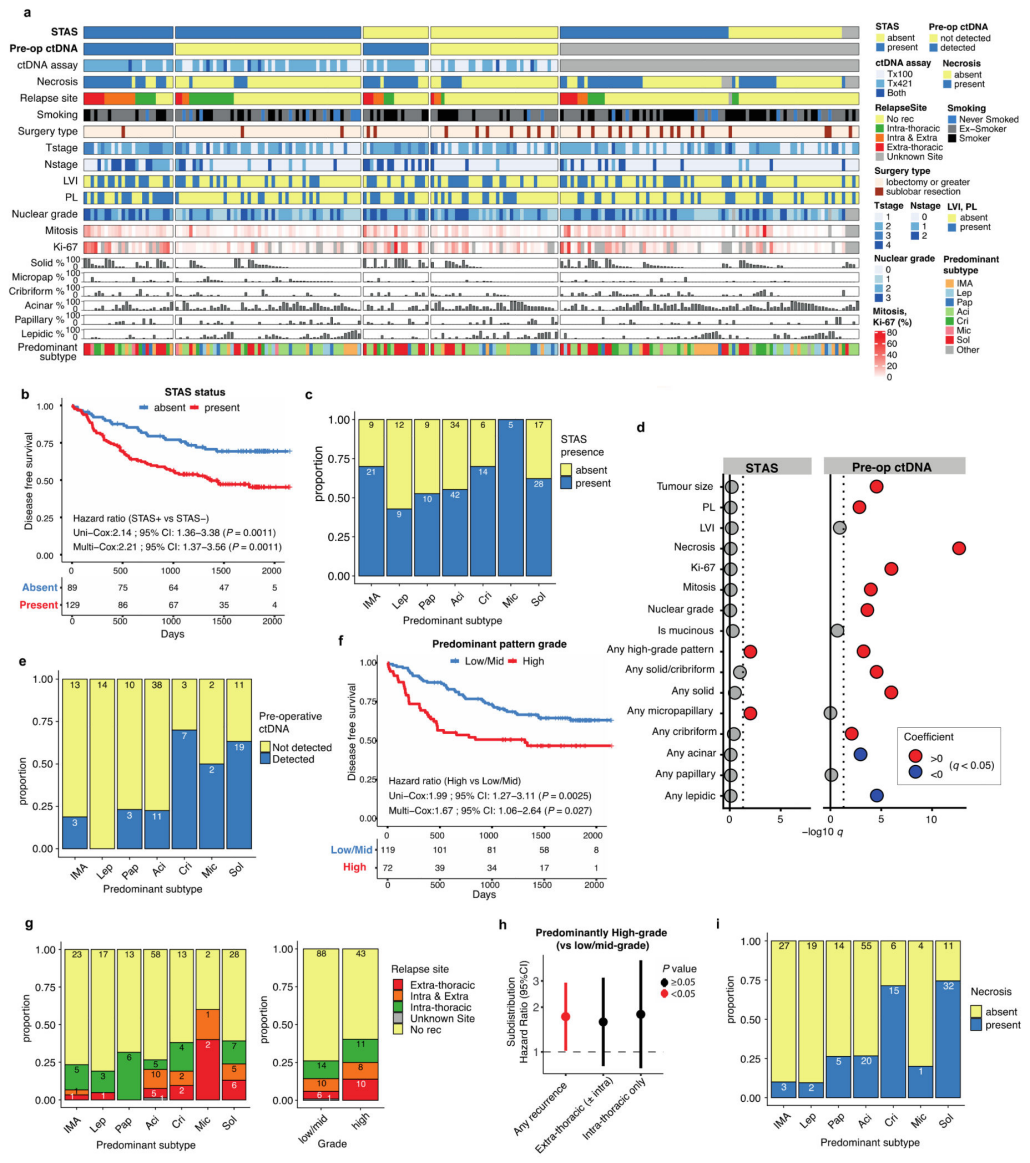
Characterisation of tumours with STAS and pre-operative ctDNA shedding **a**. Overview of the TRACERx421 LUAD cohort, ordered by the positivity of STAS, pre-operative ctDNA detection, and the site of the relapse (n = 223). Patients with synchronous primary lung cancers were excluded. Colloid and fetal adenocarcinomas are included (predominant sutbype = Other). Each column represents each patient. IMA, invasive mucinous adenocarcinoma; LVI, lymphovascular invasion; PL, pleural invasion. Tumours that did not relapse before death or the development of a new primary cancer are treated as no recurrence (No rec). **b**. Kaplan-Meier curve of disease-free survival, comparing STAS present vs absent. Numbers at risk are described at the bottom. For the patients with multiple tumours, only patients having LUAD as the most advanced tumour were included in the analysis. Hazard ratio (HR) adjusted for age, stage, pack-years, surgery type, and adjuvant therapy is shown. **c**. STAS positivity across predominant subtypes of the primary tumour. **d.** Histopathological features associated with STAS positivity (**left**) and pre-operative ctDNA detection (**right**). Negative log of the *q* values (Benjamini-Hochberg method) in univariable logistic regression analyses are presented. Vertical dotted lines represent *q* = 0.05, and variables with *q* < 0.05 are presented in points with colours which represent the direction of the correlation. **e**. Pre-operative ctDNA positivity across predominant subtypes of the primary tumour. **f**. Kaplan-Meier curve of disease-free survival, comparing patients with predominantly high-grade tumours vs low/mid-grade tumours. Numbers at risk are described at the bottom. Hazard ratio (HR) adjusted for age, stage, pack-years, surgery type, and adjuvant therapy is shown. **g.** Frequency of the relapse site (intra- and/or extra-thoracic) across predominant subtypes (**left**) and grades of the predominant subtype (**right**) of primary tumour. Tumours that did not relapse before death or the development of a new primary cancer are treated as no recurrence (No rec). **h.** Relapse-site specific (subdistribution) hazard ratio for predominantly high-grade tumours compared with low/mid-grade tumours in all LUADs, adjusted for age, stage, pack-years, surgery type, and adjuvant therapy (n = 185). *P* < 0.05 are described in red (unadjusted for FDR). **i**. Positivity of necrosis across predominant subtypes of the primary tumour.

A particular case of interest involved a diagnosis of primary lepidic predominant (non-mucinous) adenocarcinoma (CRUK0296) which was found to have a pure lepidic intrapulmonary metastasis during follow-up. After primary resection, the patient developed a subsequent tumour in the contralateral lung, which was surgically resected, and a diagnosis of a pure lepidic pattern LUAD was made. This was deemed a second primary lung cancer due to its 100% lepidic pattern, however, through TRACERx genomic profiling both tumours were found to be clonally related indicating that this was an intrapulmonary metastasis (Extended Data Fig. 6a-d). Notably, pure lepidic intrapulmonary metastasis were not identified in a previous study of 23 intrapulmonary LUAD metastases, although both the primary and metastasis showed part-lepidic patterns in 14 (61%) of the cases^21^. Furthermore, the primary tumour demonstrated evidence of ‘spread through air spaces’ (STAS) (Extended Data Fig. 6c), and the presence of a confirmed metastatic lesion that is purely lepidic, and therefore without stromal invasion, supports the hypothesis that free-floating cells are capable of seeding distant tumours through the airways by aerogenous spread. There were five additional patients in whom lung metastases were sequenced upon primary surgery sampling and/or during follow-up, and in which the primary tumour demonstrated positive STAS. Phylogenetic analysis revealed late divergence in all cases (Extended Data Fig. 6e), which was defined by the divergence of metastatic clone occurred after the last complete clonal sweep within the primary tumour^11^. Although the predominant primary tumour subtype was unrelated to the timing of metastatic divergence (Extended Data Fig. 6f), STAS positivity was significantly associated with late divergence (*P* = 0.019, Fisher’s exact test) (Extended Data Fig. 6g), suggesting that the ability to metastasise through the airway may be a late event during LUAD evolution, or that tumours acquiring the ability to metastasise through the airway early in their evolution may be rare in our current surgical cohort. Overall, these findings prompted a more detailed analysis of the relationship between histological pattern, STAS, and patient outcome, including the site of relapse (Extended Data Fig. 7a).

### Impact of tumour morphology upon site and risk of recurrence

The morphological feature of STAS is defined as free-floating tumour cells, or tumour cell clusters, in air spaces beyond the boundary of the tumour, and is known to be associated with intra-thoracic recurrence in limited (sublobar) resections in stage I LUAD ^3,22,23^. Others have reported an association with STAS and poor prognosis in more advanced stage LUAD^24,25^ as well as in non-LUAD histologies^26,27^. In a multivariable analysis of the TRACERx 421 LUAD cohort, disease-free survival (DFS) of STAS positive cases was shorter than STAS negative cases (HR=2.2 [95%CI 1.4-3.6], adjusted for age, stage, pack-years, surgery type, and adjuvant therapy) (Extended Data Fig. 7b).

**Extended Data Fig. 8 |:**
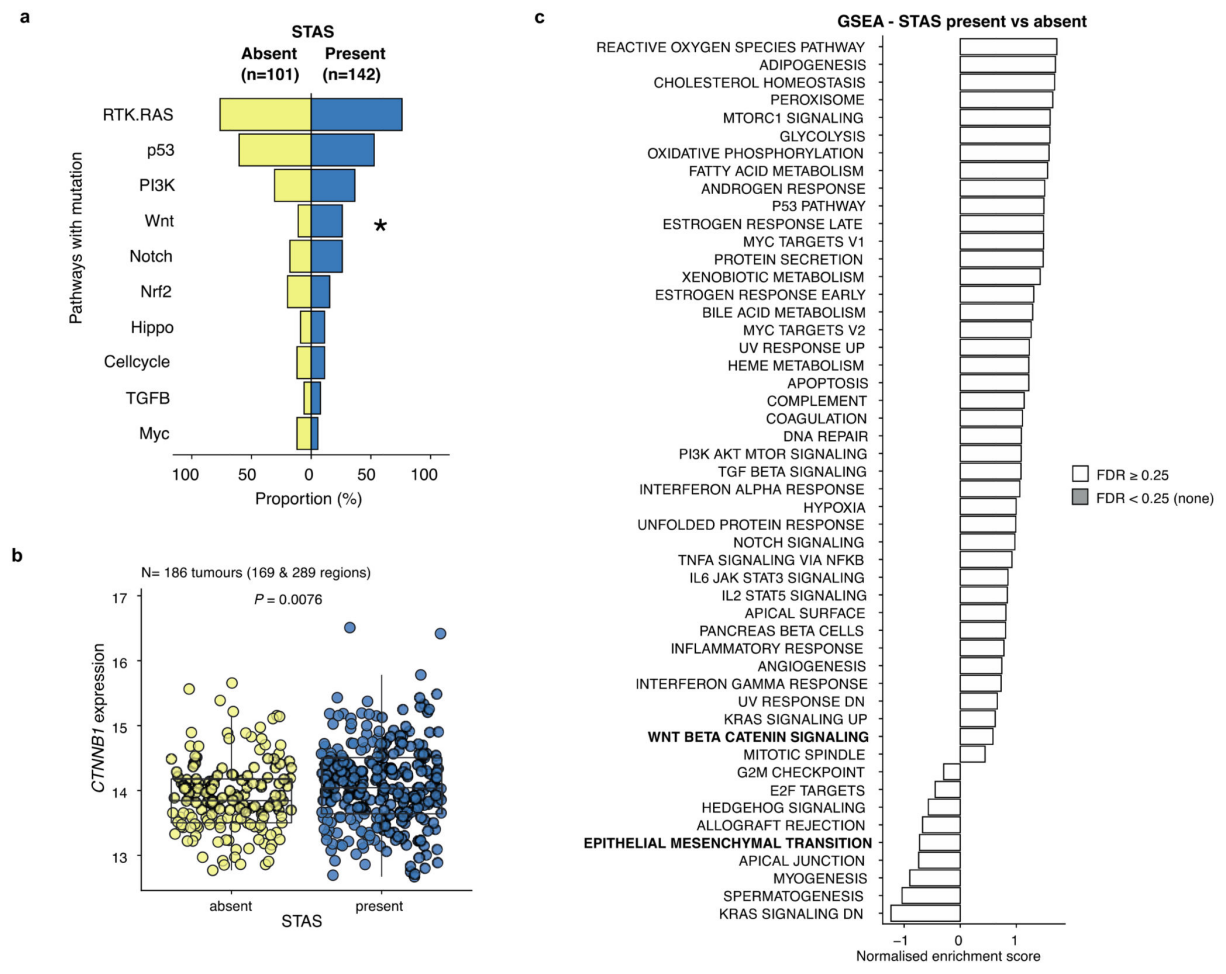
Genomic and transcriptomic analyses of STAS in LUAD **a.** Frequency of driver mutations in 10 canonical oncogenic signalling pathways^49^ in STAS present and absent tumours. P values (Fisher’s exact test) were corrected for multiple testing according to Benjamini-Hochberg (BH) and the asterisk indicates *q* value range * *q* < 0.05. **b**. Comparison of *CTNNB1* gene expression (variance stabilisation normalised count) between STAS positive and negative tumours. Centre line, median; box limits, upper and lower quartiles; whiskers, 1.5× interquartile range. *P* value was calculated using a linear mixed effect model, with each tumour as a random effect. **c.** Gene set enrichment analysis of Hallmark gene sets between STAS positive and negative tumours. Normalised enrichment score is displayed on the x-axis and indicates the enrichment for a given gene set. None of the gene sets showed *q* < 0.25 (BH method).

STAS positivity was associated with the presence of high-grade pattern in each tumour (*q* = 0.0096, univariate logistic regression, ANOVA, FDR adjusted; Methods), and was associated with micropapillary patterns (*q* = 0.0096) (Extended Data Fig. 7c-d), as described in other cohorts^3,24^. Immunohistochemical nuclear beta-catenin positivity and an epithelial to mesenchymal transition (EMT) phenotype has previously been shown to be associated with STAS^28^. Driver mutations in the Wnt pathway were enriched in STAS positive tumours, (*q* = 0.033, Fisher’s exact test, FDR adjusted) (Extended Data Fig. 8a), and the bulk tumour transcriptomic profiles showed higher *CTNNB1* gene expression (*P* = 0.0076, linear mixed effect model, ANOVA) (Extended Data Fig. 8b). However, we did not observe enrichment of EMT pathway or Wnt-beta-catenin signalling gene expression modules in STAS positive tumours (Extended Data Fig. 8c), potentially due to the difficulty of capturing phenotypic differences related to STAS using bulk transcriptomic data.

**Fig. 4 |:**
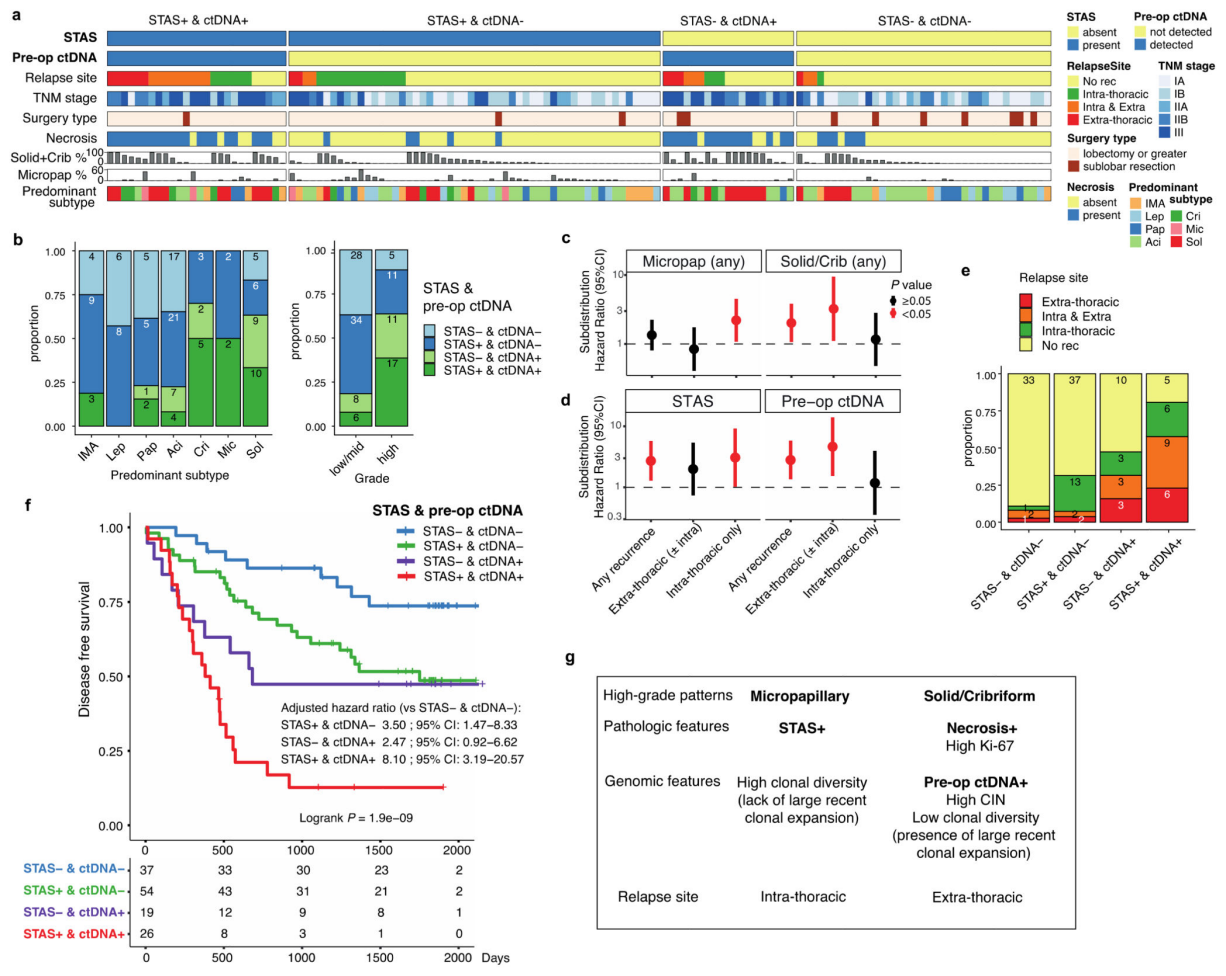
Impact of tumour morphology upon site and risk of recurrence **a**. Overview of the TRACERx 421 LUAD cohort with STAS assessment and pre-operative ctDNA data (n = 136 patients), excluding the patients with synchronous primary lung cancers. Each column represents each patient. IMA, invasive mucinous adenocarcinoma. Tumours that did not relapse before death or the development of a new primary cancer are treated as no recurrence (No rec). **b.** Frequency of STAS and pre-operative ctDNA positivity across predominant subtypes (**left**) and grades of the predominant subtype (**right**) of primary tumour. **c-d**. Relapse-site specific (subdistribution) hazard ratio (HR) for (**c**, **left**) the presence of micropapillary pattern and (**c**, **right**) the presence of solid and/or cribriform patterns (n = 215), and (**d, left**) the positivity of STAS and (**d**, **right**) pre-operative ctDNA detection in patients with pre-operative ctDNA data (n = 131). HR were adjusted for age, stage, pack-years, surgery type, and adjuvant therapy using Fine-Gray regression model. *P* < 0.05 are shown in red (unadjusted for FDR). **e**. Frequency of the relapse site (intra- and/or extra-thoracic), stratified by the positivity of STAS and pre-operative ctDNA detection. Tumours that did not relapse before death or the development of a new primary cancer are treated as no recurrence (No rec). **f.** Kaplan–Meier curves of disease-free survival, split by the positivity of STAS and pre-operative ctDNA detection. Hazard ratios were adjusted for age, stage, pack-years, surgery type, and adjuvant therapy. The number of patients in each group for every time point is indicated below the time point. **g**. Summary of the findings related to high-grade patterns, pathologic and genomic features, and relapse site. Factors with prognostic impact investigated in the study are highlighted in bold.

The presence of pre-operative ctDNA is known to be associated with increased risk of relapse in LUAD^29^. In our companion manuscript, we show the presence of preoperative ctDNA is particularly associated with extra-thoracic recurrence^30^, which may reflect the increased risk of hematogenous metastatic dissemination. In a subset of the LUAD cohort excluding the patients with synchronous primary lung cancers (136/242 patients), pre-operative ctDNA detection from two assays (53 patients with an assay previously reported by our group in Abbosh et al^9^, and 90 patients with an assay reported in our companion manuscript^30^, including 7 patients analysed in both assays), and STAS status were integrated to compare the biological features of these two prognostic indicators in relation to the risk and site of metastasis (Fig. 4a). Patients with both STAS positivity and pre-operative ctDNA detection had primary tumours enriched for predominantly high-grade patterns (*P* = 7.0 × 10^-5^, Fisher’s exact test) (Fig. 4b).

**Extended Data Fig. 9 |:**
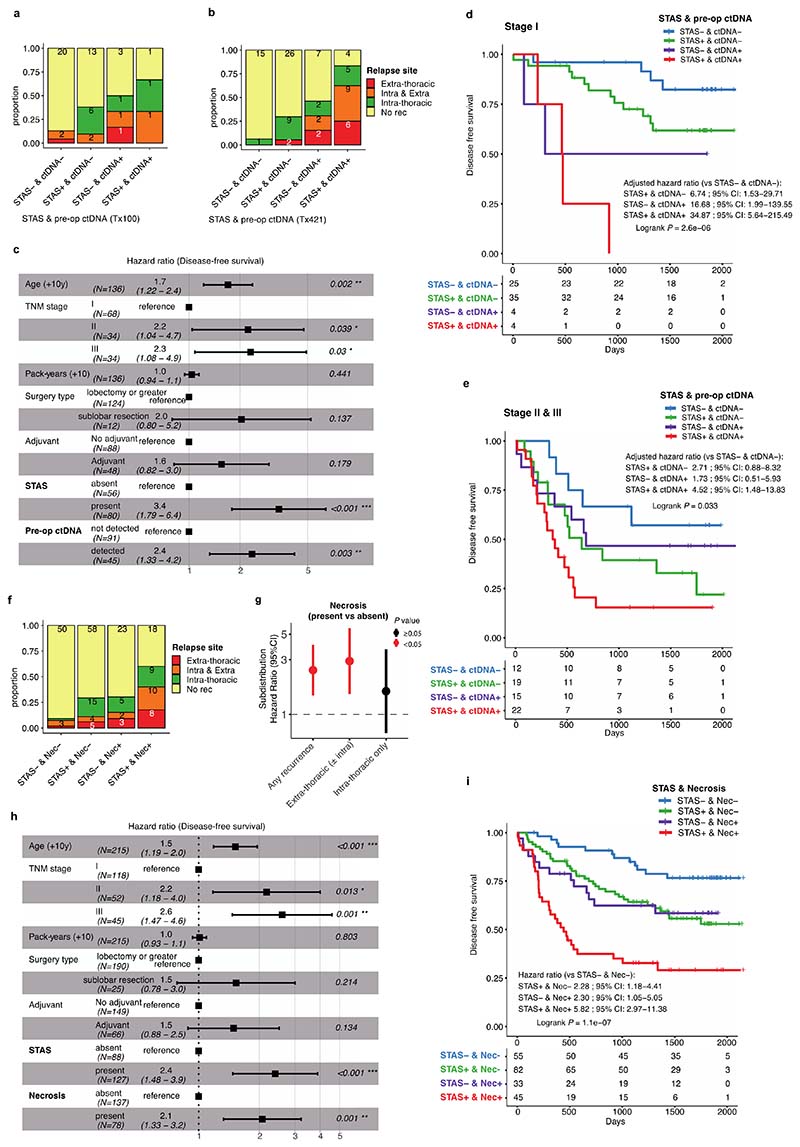
Impact of STAS, pre-operative ctDNA positivity, and necrosis on sites and risk of recurrence **a-b**. Frequency of the relapse site (intra- and/or extra-thoracic), stratified by the positivity of STAS and pre-operative ctDNA detection. Pre-operative ctDNA data were based on (**a**) the assay previously reported by Abbosh et. al^9^ (Tx100 cohort) and (**b**) the assay reported in our companion manuscript^30^ (Tx421 cohort), including 7 patients who underwent both assays in each cohort. Tumours that did not relapse before death or the development of a new primary cancer are treated as no recurrence (No rec). **c.** Positivity of STAS and pre-operative ctDNA detection are incorporated with other tumour and clinical characteristics in a multivariable Cox proportional hazards model (disease-free survival). Hazard ratios (HRs) of each variable with 95% confidence intervals (CIs) are shown on the horizontal axis. **d-e**. Kaplan–Meier curves of disease-free survival, split by the positivity of STAS and pre-operative ctDNA detection in (**d**) stage I patients and (**e**) stage II & III patients. HRs were adjusted for age, stage, pack-years, and adjuvant therapy. Surgery type was also added as a covariate for stage I patients but not for stage II & III patients, because only 1 patient underwent sublobar resection in stage II & III patients. The number of patients in each group for every time point is indicated below the time point. **f**. Frequency of the relapse site (intra- and/or extra-thoracic), stratified by the presence of STAS and necrosis in all LUADs. **g**. Relapse-site specific (subdistribution) HR for positivity of necrosis in all LUADs, adjusted for age, stage, pack-years, surgery type, and adjuvant therapy (n=211). *P* < 0.05 are shown in red (unadjusted for FDR). **h.** Positivity of STAS and necrosis are incorporated with other tumour and clinical characteristics in a multivariable Cox proportional hazards model for disease-free survival. HRs of each variable with 95% CIs are shown. **i**. Kaplan–Meier curve of disease-free survival, split by the positivity of STAS and the presence of necrosis. HRs were adjusted for age, stage, pack-years, surgery type, and adjuvant therapy. The number of patients in each group for every time point is indicated below the time point.

Detection of pre-operative ctDNA was associated with the presence of high-grade patterns (*q* = 5.4 × 10^-4^, univariate logistic regression, ANOVA, FDR adjusted; Methods), in particular solid (*q* = 1.0 × 10^-6^) and cribriform (*q* = 0.008) patterns, and a lack of lepidic (*q* = 2.7 × 10^-5^) and acinar patterns (*q* = 0.0011) (Extended Data Fig. 7d-e), consistent with previously reported radiological characteristics in ctDNA shedding tumours^29^. As described in an earlier TRACERx cohort, histological evidence of necrosis (*q* = 2.2 × 10^-13^), tumour size (*q* = 2.8 × 10^-5^), Ki-67 fraction (*q* = 9.9 × 10^-7^), mitotic index (*q* = 1.1 × 10^-4^), degree of nuclear pleomorphism (nuclear grade, *q* = 2.3 × 10^-4^), and the presence of pleural invasion (*q* = 0.0014) were associated with pre-operative ctDNA detection^9^, but not with STAS positivity (Extended Data Fig. 7d). Whilst predominantly high-grade tumours were associated with shorter DFS than predominantly low/mid-grade tumours (HR = 1.7 [95%CI 1.1-2.6], multivariable Cox regression) (Extended Data Fig. 7f), predominance of high-grade pattern was not significantly associated with relapse site (Extended Data Fig. 7g-h). In contrast, the presence of micropapillary pattern was associated with intra-thoracic-only recurrence (subdistribution HR = 2.3 [95%CI 1.1-4.6], multivariable Fine-Gray regression) and the presence of solid and/or cribriform patterns was associated with extra-thoracic recurrence (subdistribution HR = 3.2 [95%CI 1.1-9.4]) (Fig. 4c, Extended Data Fig. 7i), consistent with findings in stage I LUADs reported previously^31,32^. Similarly, STAS positivity was associated with increased risk of intra-thoracic-only recurrence (subdistribution HR = 3.0 [95%CI 1.0-9.1], multivariable Fine-Gray regression), but not extra-thoracic recurrence (subdistribution HR = 2.0 [95%CI 0.7-5.4]). Although it is worth noting that our cohort may be underpowered to detect the risk of extra-thoracic recurrence in STAS positive tumours, which has been reported previously in a larger cohort^24,25^. Pre-operative ctDNA detection was associated with extra-thoracic recurrence (subdistribution HR = 4.6 [95%CI 1.5-13.8], multivariable Fine-Gray regression) but not intra-thoracic-only recurrence (subdistribution HR = 1.2 [95%CI 0.3-3.9]) (Fig. 4d), as reported in our companion manuscript^30^. Of note, STAS was detected in 17 out of 21 patients (81%) who had disease relapse despite having undetectable pre-operative ctDNA, which was significantly higher than STAS detection in tumours with undetectable pre-operative ctDNA and no subsequent relapse (37/70, 52.8%) (*P* = 0.024, Fisher’s exact test) (Fig. 4e, Extended Data Fig. 9a-b).

Patients with both STAS positivity and pre-operative ctDNA detection had an increased risk of disease relapse compared to patients in whom neither were detected (HR = 8.1 [95%CI 3.2-20.6], multivariable Cox regression) (Fig. 4f). Both STAS positivity and preoperative ctDNA detection were independent predictors of prognosis in a multivariable analysis that included age, stage, pack-years, surgery type, and adjuvant therapy (STAS, HR = 3.4 [95%CI 1.8-6.4]; pre-operative ctDNA, HR = 2.4 [95%CI 1.3-4.2], multivariable Cox regression) (Extended Data Fig. 9c). These results suggest that whilst STAS positivity and pre-operative ctDNA detection are both associated with disease recurrence, the underlying biology of the metastatic process in tumours with each of these characteristics is distinct. Furthermore, the combination of STAS positivity and pre-operative ctDNA detection has the potential to identify patients with an increased risk of relapse during follow-up, independent of TNM staging, and is therefore of potential clinical utility (Extended Data Fig. 9d-e).

**Extended Data Fig. 10 |:**
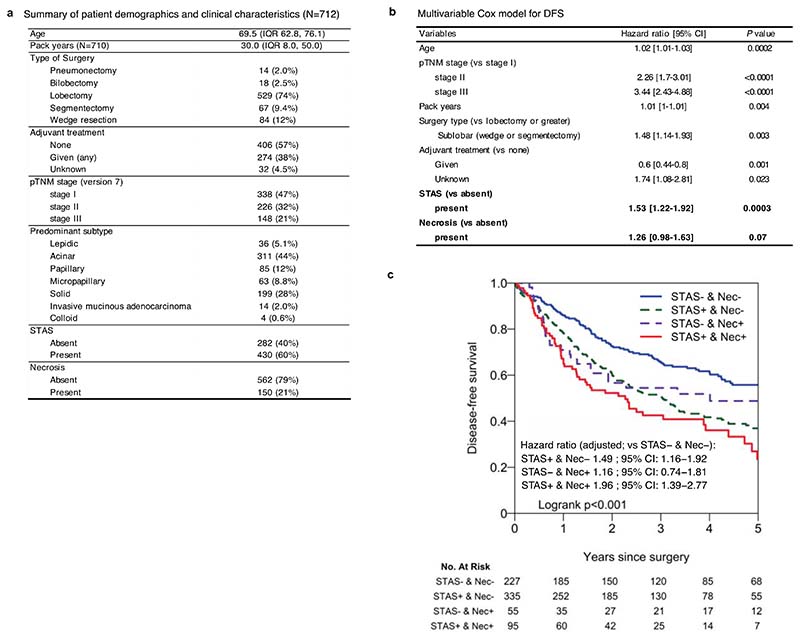
External validation of the impact of STAS and necrosis on disease-free survival **a**. Summary of patient demographics and clinical characteristics of the Memorial Sloan Kettering Cancer Center cohort (n = 712). **b.** Positivity of STAS and necrosis are incorporated with other tumour and clinical characteristics in a multivariable Cox proportional hazards model of disease-free survival. **c.** Kaplan–Meier curve of disease-free survival, split by the positivity of STAS and the presence of necrosis (n = 712). Hazard ratios were adjusted for age, stage, pack-years, surgery type, and adjuvant therapy. The number of patients in each group for every time point is indicated below the time point.

Finally, since histological evidence of necrosis was more significantly associated with pre-operative ctDNA detection than any other histological feature (Extended Data Fig. 7d), we tested whether necrosis could be used as a proxy for pre-operative ctDNA detection. The presence of necrosis was associated with solid and cribriform predominant tumours (solid/cribriform vs others, *P* = 3.7 × 10^-13^, Fisher’s exact test) (Extended Data Fig. 7i) and an increased risk of extra-thoracic recurrence (subdistribution HR = 2.9 [95%CI 1.5-5.6], multivariable Fine-Gray regression), but not intra-thoracic-only recurrence (subdistribution HR = 1.6 [95%CI 0.7-3.6]) (Extended Data Fig. 9f-g). As a combined measure, these two histological features remained significant independent predictors of outcome in a multivariable analysis (STAS, HR = 2.4 [95%CI 1.5-3.9]; necrosis, HR = 2.1 [95%CI 1.3-3.2], multivariable Cox regression) (Extended Data Fig. 9h). The combination of STAS and necrosis demonstrated that patients with tumours positive for both had an increased risk of disease relapse (HR = 5.8 [95%CI 3.0−11.4] versus patients negative for both, multivariable Cox regression) (Extended Data Fig. 9i). A similar result was observed in a larger independent external cohort of surgically resected stage IB-IIIA LUADs (n = 712, HR = 2.0 [95%CI 1.4−2.8] versus patients negative for both, multivariable Cox regression) (Extended Data Fig. 10a-c). The combination of STAS and necrosis may therefore have clinical value in predicting metastatic risk in patients in the absence of pre-operative ctDNA sampling and analysis.

## Discussion

Adenocarcinoma of the lung is a morphologically heterogeneous disease both at the inter- and intra-tumoural level. This study of the diverse growth pattern subtypes in prospectively collected multi-region and longitudinal samples, matched to the regional genomic and transcriptomic analysis performed in the TRACERx study, reveals novel insights into the molecular characteristics of LUAD subtypes, which may contribute to our understanding of tumour evolution and ability to predict clinical behaviour and stratify risk. Of the high-grade patterns, solid and cribriform are associated with pre-operative ctDNA detection and necrosis, are reflecting high chromosomal instability, proliferation and recent subclonal expansion, and are related to extra-thoracic recurrence. Micropapillary pattern, the other high-grade pattern, is associated with positivity for STAS and higher clonal diversity, and is related to intra-thoracic recurrence (Fig. 4g). Although the inter-correlations of these pathologic, genomic and prognostic features are highlighted in this study, their correlation is still limited and genomic and histological subtypes are therefore not entirely interchangeable and as such histological profiling cannot be supplanted by genomic data, or vice versa. Here we show the clinical utility of combining the presence of STAS and pre-operative ctDNA data derived from integrated genomic analysis of tumour and serum. The combination of histological and genomic information may therefore be used complementarily to better predict the likelihood and site of the recurrence, and to optimise (neo-)adjuvant treatment indication and post-operative screening modality.

Multi-region sampling and haplotype-specific SCNA analysis allows for the detection of clonal and subclonal SCNAs and mutations with higher sensitivity than previously reported in single tumour region cohorts. It has previously been shown that predominantly high-grade tumours are associated with high TMB and fraction of genome altered by SCNA^6^. In this TRACERx study, truncal, but not subclonal, TMB was associated with the proportion of high-grade patterns at the whole tumour level. The proportion of subclonal SCNAs, a key indicator of chromosomal instability and SCNA intra-tumour heterogeneity, and the presence of a large recently expanded subclone, which is associated with metastatic potential and poor survival outcome^10^, were also identified as enriched in high-grade tumours. Notably, these findings were reliant on the analysis of multi-region sequence data available through the TRACERx study.

Multi-region data further enabled us to focus on truncal genomic events that may constrain tumour evolution toward predominantly low/mid-grade, high-grade and purely solid tumours. Co-occurence of truncal chromosome 3p and 3q loss/LOH, possibly a truncal loss of whole chromosome 3, was enriched in predominantly low/mid-grade tumours, suggesting as an evolutionary constraint associated with low/mid-grade patterns, whilst truncal chromosome arm 3q gain, especially truncal gain of focal 3q (3q21.3-3q29) encompassing several driver genes (*TERC*, 3q26.2; *PIK3CA*, 3q26.32) and tissue development- and differentiation-related genes (*SOX2*, 3q26.33; *TP63*, 3q28), as well as truncal *SMARCA4* alterations were associated with purely solid tumours. *SMARCA4* deficiency is associated with undifferentiated thoracic carcinoma^33–35^ and 3q gain is a characteristic feature of lung squamous cell carcinoma (LUSC) rather than LUAD^36^. Focal gain in chromosome 3q has also been described as a characteristic of LUSC in NSCLC^37^. This suggests that pure solid tumours, commonly classified histologically as LUAD on the basis of TTF-1 immunohistochemical staining alone, may have a distinct evolutionary trajectory from mixed pattern tumours with a solid component and harbour common genomic traits with other NSCLC histological types. Molecular profiling of undifferentiated tumours, including purely solid LUADs and non-LUAD subtypes, may improve the classification of NSCLC in relation to underlying evolutionary trajectories unique to each subtype, which may in turn inform the ability to predict treatment response and prognosis, and warrants further investigation.

The inferred transition towards higher grade patterns in primary tumour ancestor-like-to-descendant-like analysis and in seeding region-to-metastasis analysis may reflect the evolutionary trajectories adopted from lower to higher grade patterns. Of note, the inference of subclonal evolution from higher to lower grade patterns observed in a minority of primary tumours suggests some plasticity in growth patterns, which may reflect epigenetic and/or tumour microenvironmental factors, as previously suggested^38^. However, the rare observation of higher to lower grade (“downward”) transition can also be explained in other manners. Strictly speaking, one region may consist of indirect descendant clones of another region of the tumour, but not direct descendant clones. We cannot rule out the possibility that both regions in a pair that appeared as a downward transition in our analysis had in fact evolved in parallel from an ancestral low-grade region. Furthermore, we cannot exclude the possibility of over-calling downward transition technically, due to ambiguity in SCNA profiling or growth pattern annotation.

Preclinical models including other tumour types have demonstrated the impact of spatial constraints upon the pattern of tumour growth^39^. In our cohort, lymph node metastatic samples are heavily represented and this tissue type contains less structural matrix and more spatial restrictions compared with typical lung parenchyma. The shift from low/mid-grade seeding regions in the primary tumour to high-grade metastasis may therefore be partly explained by the differing microenvironment in the primary and metastatic sites.

As previously reported, both STAS and ctDNA positivity were individually associated with poor prognosis in LUAD^24,25,29^. STAS positive tumours are known to be associated with locoregional recurrence after limited resection in stage I LUAD^3,22,23^, which may reflect increased risk of non-hematogenous dissemination of tumour cells. Conversely, increased risk of extra-thoracic recurrence is observed in patients in whom pre-operative ctDNA is detected^30^, and ctDNA detection in LUAD may reflect the increased risk of hematogenous cancer cell dissemination. The presence of solid/cribriform pattern was associated with pre-operative ctDNA detection and increased risk of extra-thoracic recurrence, whilst micropapillary pattern was associated with STAS positivity and intra-thoracic recurrence. Of note, The International Association for the Study of Lung Cancer (IASLC) proposed a LUAD grading system in which high-grade pattern ≥ 20% is shown to predict poor patient outcome^5^. The increased risk of hematogenous dissemination in tumours with solid/cribriform patterns and of non-hematogenous dissemination in tumours with micropapillary pattern may explain the poor prognosis associated with all three high-grade patterns.

The differing histological, genomic, and transcriptomic profiles and patterns of recurrence in the STAS positive and pre-operative ctDNA detected patients suggest that different biological mechanisms may be involved in determining poor outcomes. The high rate of STAS positivity (81%) in patients with undetectable pre-operative ctDNA who subsequently developed disease relapse, more often within the thorax, strongly suggests STAS is a reflection of the driving mechanism of seeding metastasis in tumours that do not undergo haematogenous spread.

In this cohort, it has been possible to demonstrate that patients who are positive for both STAS and pre-operative ctDNA have particularly poor outcomes. As a combined measure, both STAS positivity and ctDNA detection have the potential to predict outcome at resection, differentiating the underlying mechanisms of metastatic dissemination following curative surgery.

The prognostic relevance of necrosis, mitotic index, and nuclear grade has been previously reported^40–43^. Here we confirmed these histological features are associated with pre-operative ctDNA shedding and therefore detection, with necrosis showing the strongest association. Necrosis could therefore represent a proxy for ctDNA detection when predicting prognosis, and, supported by validation of these combined features in a large independent cohort, these data strongly suggest clinical value for combined STAS and necrosis scoring in patients who have undergone a LUAD resection but do not have available pre-operative ctDNA data. However, in our analysis, we had limited power to analyse stage IA patients especially those with tumour size <2cm due to the requirement for multi-region sampling and the subsequent impact upon patient recruitment. Further investigation is therefore needed for these patients. Of note, the utility of combining profiles of necrosis, STAS, lymphovascular invasion, high-grade patterns (solid and micropapillary), and surgical procedure have been proposed for stage IA (<2cm) patients^44^, potentially reflecting the low positivity of each risk factors and suggesting that the risk profiling by STAS and necrosis alone may be insufficient for stage IA (<2cm) tumours.

Whilst the tumour growth patterns in this cohort were scored in line with the WHO classification, LUAD morphologies represent a spectrum and there is inter-observer variability when assessing histological patterns, dependent upon the ability to accurately distinguish patterns^45^. In this study, a comprehensive central pathology review by multiple pathologists and the patient cohort size were mitigating factors against these limitations. Furthermore, multi-region sampling of matched primary and metastatic tumours in addition to regional sequencing performed in TRACERx enabled the integration of tumour morphology with genomic and transcriptomic phylogenetic analyses.

Overall, these data reveal novel aspects of the underlying biology of high-grade LUAD patterns and their increased metastatic potential, as well as detailing the differing mechanisms of metastasis adopted by tumours with specific growth patterns. Given that variable differentiation and morphology is a common feature of many cancers, the characteristics of high-grade disease described here may be relevant to other tumour types, and at the very least suggest the need for their exploration. Furthermore, these data demonstrate the relevance of measures relating to STAS, necrosis and ctDNA detection in defining patient risk stratification and predicting prognosis.

## Methods

### The TRACERx 421 cohort, sample collection, and DNA/RNA sequencing

All patients were enrolled into the TRACERx study (https://clinicaltrials.gov/ct2/show/NCT01888601, approved by an independent research ethics committee, 13/LO/1546), with multi-region sampling of tumours, DNA and RNA extraction, whole exome sequencing (WES) and RNA sequencing as previously described ^1,2^. The first 421 patients, which constitute the first half of the originally scheduled full cohort of the clinical trial, are included in the TRACERx 421 cohort. Inclusion/exclusion criteria of the clinical study, clinical data acquisition, and bioinformatic processing of DNA and RNA sequencing data, including fusion gene analysis and pre-operative ctDNA analysis, were performed as described in our companion manuscripts^3–6^.

### Histopathological assessment

#### Central histopathological review

The diagnostic slides from all lung adenocarcinoma (LUAD) cases in the cohort were requested from the local pathology departments, scanned using a Hamamatsu Nanozoomer S210 slide scanner at 40x scanning magnification and retained within a central digital histology archive. In a small number of cases, full diagnostic slides were not available and therefore pathology review was conducted using a combination of a single representative diagnostic slide and regional TRACERx tissue samples. Full diagnostic slides were used for central pathology review to confirm tumour subtype and to generate adenocarcinoma growth pattern fractions following review by central study pathologists (Extended Data Fig. 1k). Tumours were categorised into six growth patterns - the five patterns currently defined in the WHO classification^7^, with the addition of a cribriform pattern which has elsewhere been included as part of the acinar growth pattern subtype (Fig S1A-H). As per standard clinical diagnostic practice and current guidance, we labelled each tumour using the predominant histological subtype according to the proportions of each growth pattern. Invasive mucinous adenocarcinoma (including mixed invasive mucinous and nonmucinous adenocarcinoma) was labelled as a separate entity, in line with the WHO classification, though pattern proportions of the six growth patterns were still ascribed. For 28/242 patients whose full diagnostic slides were not available, pattern proportion was based upon local histopathology reporting, provided this matched broadly with the appearances of any available material at central review. Any discrepancy in tumour type between the clinical pathology report and central review was subject to additional expert review for a final definitive diagnosis. The presence of STAS was defined as previously described^8,9^.

#### Nuclear grade and mitotic index

The nuclear grade was determined based upon nuclear size in the highest grade area of the tumour, as previously described^10^. The mitotic index was determined from diagnostic material from the area of the tumour with the highest mitotic activity. The count was performed over a 2.4mm^2^ area of tumour on scanned slides, equal to the area of 10 high-power microscopic fields used in previous LUAD grading studies^11^.

#### Regional growth pattern annotation

Where regional fresh tissue samples were sufficient, these samples were split between fresh frozen tissue for DNA and RNA extraction, and formalin-fixed paraffin-embedded (FFPE) tissue to allow histological assessment of the sequenced regions. This regional histology was used to generate regional growth pattern data in lung adenocarcinoma and regional stromal TIL scores for the entire cohort.

#### Definition of tumour growth pattern homogeneity

A tumour was defined as morphologically homogeneous when the tumour met both of the following criteria: 1) predominant subtype was dominating 90% or more of the tumour area in diagnostic slides and 2) predominant subtype was dominating 90% or more of the annotated regional patterns.

#### Growth pattern annotation in the metastatic sample

In metastatic disease, where possible, the metastatic tumour was sampled for sequencing analysis. If sufficient tissue was available for histological assessment, growth pattern was also characterised in lung adenocarcinoma samples. Biopsies that were too limited for growth pattern annotation or were too dissociated, such as some aspirated samples, were excluded from the analysis.

#### PD-L1 and Ki-67 immunohistochemical staining

PD-L1 (22C3 clone) and Ki-67 (MIB-1 clone) immunohistochemistry were performed on a single representative diagnostic FFPE tissue block from the resection specimen using a Link48 Autostainer (Agilent) for PD-L1 and a Bond- III Autostainer (Leica Biosystems) for Ki-67, according to the manufacturer’s instructions. Fractions of positively stained tumour cells (cancer cells) were scored manually by a pathologist in line with clinical guidelines.

#### Pathology TIL estimates

TILs were estimated from haematoxylin and eosin (H&E) stained slides using established international guidelines, developed by the International Immuno-Oncology Biomarker Working Group, as described in the previous reports^2,12,13^. In brief, the relative proportion of stromal area to tumour area was determined from the pathology slide of a given tumour region. TILs were reported for the stromal compartment (= percent stromal TILs). The denominator used to determine the percent stromal TILs was the area of stromal tissue. Therefore percent stromal TILs equalled the area occupied by mononuclear inflammatory cells over the total intratumoral stromal area rather than the fraction of total stromal nuclei that represent mononuclear inflammatory cell nuclei. This method has been demonstrated to be reproducible among trained pathologists^14^. The International Immuno-Oncology Biomarker Working Group has developed a freely available training tool to train pathologists for optimal TIL assessment on H&E slides (www.tilsincancer.org).

### TRACERx analytical pipeline and orthogonal method for SCNA profiling and clonality inference

#### SCNA profiling

In the TRACERx WES pipeline, copy number segmentation data were produced using ASCAT^15^ and then a multi-sample somatic copy number alteration (SCNA) estimation approach^3,16^ was applied in which single nucleotide polymorphisms (SNPs) are phased onto paternal and maternal alleles using samples with allelic imbalance and detectable B-allele-frequency (BAF) separation. This approach allowed copy number aberrations present in one region to be tested for in other regions, and enabled us to more accurately profile copy number states in low purity tumour regions. To determine genome-wide copy number gain and loss events, copy number data for each sample was divided by the sample mean ploidy, and log2 transformed. Amplification, gain, and loss thresholds were defined as log2 (4/2), log2(2.5/2), and log2(1.5/2), respectively. LOH was defined as a floating point copy number of the minor allele of <0.5. As discussed previously^1^, our pipeline may be under-calling homozygous deletions, due to the nature of the relatively low resolution of the WES data (restricted to exonic regions) making it difficult to call very focal events. Therefore, we excluded homozygous deletions from the analysis.

#### Clonality inference

In the TRACERx WES pipeline, mutations were classified as truncal or subclonal using a modified version of PyClone(v0.13.1)^17^. Several additional steps were then carried out during phylogenetic tree building to avoid overcalling subclonality and inappropriately increasing the reported degree of tumour heterogeneity^1,3^. For copy number amplification, if at least one region showed an amplified mean copy number, we called the amplification truncal if all other regions showed a copy number gain of ploidy +1 copy. The gene would be called subclonally amplified if at least one region showed no gain, or if a truncal event overlapped with SCNAs that disrupt the same genomic region but affect different parental alleles within separate tumour subclones, what we called a mirrored subclonal allelic imbalance in a previous publication^1^. Truncal/subclonal chromosomal arm gain and loss were defined on a per tumour basis, by requiring at least one region to show at least 98% gain or loss of the arm. Truncal arm gain or loss was then called if the same chromosomal arm showed at least 75% gain or loss across all remaining regions. Subclonal arm gain or loss was called if at least one region showed less than 75% gain or loss of the chromosomal arm or if a chromosomal arm was subject to mirrored subclonal allelic imbalance^1^.

#### Orthogonal methods

As an orthogonal method for SCNA profiling, we used the default output of Sequenza^18^ for each sample. As a default output, Sequenza returns integer copy numbers for major and minor alleles. LOH was defined as integer copy number = 0 for the minor allele and >0 for the major allele. As an alternative method to call clonality for mutations and SCNAs, we called any ubiquitous events truncal (e.g. amplification observed in all regions per tumour), and any non-ubiquitous events subclonal (e.g. one region with amplification but all other regions were gains).

### Enumeration of chromosomal complexity, clonal architecture, and intra-tumour heterogeneity

#### Weighted genome instability index (wGII score)

The wGII score was calculated as the proportion of the genome with aberrant copy number relative to the median ploidy, weighted on a per chromosome basis^19^.

#### Fraction of genome subject to loss of heterozygosity (FLOH score)

The FLOH score was defined as the proportion of the genome subject to loss of heterozygosity.

#### % subclonal SCNA (= SCNA-ITH)

The percentage of the genome subject to subclonal SCNA events was divided by the percentage of the genome involved in any SCNA event in each tumour^1,3^.

#### % subclonal TMB (= Mut-ITH).

The number of mutations estimated to be subclonal was divided by the total number of mutations classified as either truncal or subclonal after phylogenetic tree building in each tumour^1,3^.

#### Subclonal TMB (clonal in ≥ 1 region) (‘illusion of clonality’-type subclonal mutation burden)

Mutational clusters used for phylogenetic tree building were determined to be subclonal or clonal within every region by testing whether cancer cell fraction (CCF, fraction of cancer cells harbouring the cluster of mutations) was significantly lower than 1. Subclonal TMB of regionally clonal mutations in ≥ 1 region but not in all regions (i.e. subclonal at tumour level), previously described as ‘illusion of clonality’^1,3^ mutations because they may appear as clonal when only one region is sampled per tumour^1^, was calculated for each tumour.

#### Subclonal diversity within the tumour region

First, cancer cell clone proportions, namely what percentage of the cells in that region come from each clone, were calculated using the cancer cell fraction of each mutational cluster that was used for phylogenetic tree construction. Then, the Shannon diversity index of the clone proportions was calculated to give the subclonal diversity of each region. Minimum subclonal diversity within the tumour region was used to represent the subclonal diversity per tumour^3^.

#### Recent subclonal expansion score

A recent subclonal expansion score per tumour, reflecting the size of the largest recent subclonal expansion within each tumour region, was calculated as follows^3^. First, for each tumour phylogenetic tree, the terminal nodes on the tree (i.e. leaf nodes) were identified. Then for each tumour region, the maximum CCF of any of these leaf nodes was identified. Lastly, as a tumour level metric, the subclonal expansion score was calculated by taking the maximum across the regional scores, therefore describing the maximal size of the most recent subclone expansion in each tumour.

### Determinants of predominant subtype

#### Truncal genomic alterations associated with predominantly high-grade or low/mid-grade tumours

To investigate the truncal genomic alterations associated with predominantly high-grade pattern tumours, we first compiled recurrent truncal events observed in more than 5% of the tumours and in at least 10 tumours in either predominantly high-or low/mid-grade tumours in the TRACERx 421 LUAD cohort. The compiled list was composed of 13 truncal driver alterations (7 mutations, 6 amplifications) and 31 truncal chromosomal arm SCNAs (8 gains and 23 loss/LOHs) (Extended Data Fig. 2a-c). Logistic regression analysis was performed to construct a model to distinguish between tumours with predominantly high-grade and low/mid-grade. Specifically, we constructed an initial model with the presence/absence of these 44 truncal events as explanatory variables. Stepwise model simplification was performed using the stepAIC function (MASS (7.3-54) R package). The final model was composed of 11 truncal genomic alterations, of which 7 were determined to be significantly independent variables (Fig. 1d). The results were consistent with the results when only truncal alterations observed in at least 10% of the cohort were included in the analysis, when an orthogonal tool (Sequenza^18^) for SCNA profiling was applied, or when wGII was added to the final model as a covariate to control for general genomic instability.

#### Differential copy number analysis of driver genes and chromosomal arms between predominantly high-grade and low/mid-grade tumours

To capture SCNAs with a frequency in the cohort of lower than 5%, we also compared the ploidy-adjusted copy number of driver genes and chromosomal arms between predominantly high-grade and low/mid-grade tumours. To account for multi-region input from a single tumour, a linear mixed effect model was applied (response variable = ploidy adjusted copy number of each SCNA, fixed effect = predominant subtype (high-vs low/mid-grade), random effect = tumour ID), using the nlme (3.1-153) R package. *P* values were adjusted for multiple comparisons using the Benjamini-Hochberg (BH) method. The results were consistent with the results when Sequenza^18^ was applied for SCNA profiling, or when wGII was added to the linear mixed effect model as a covariate.

#### Mutual exclusivity and co-occurrence of truncal genomic alterations associated with predominantly high-grade or low/mid-grade tumours

To determine significantly mutually exclusive and co-occurring (me-co) relationships between recurrent truncal genomic alterations observed in more than 5% of the tumours in the TRACERx 421 LUAD cohort, the Rediscover (0.3.0) R package, which applies statistical analysis based on the Poisson-Binomial distribution to take into account the alteration rate of genes and samples, was implemented^20^. The truncal events observed in at least 10 tumours in either predominantly high- or low/mid-grade tumours were included in the analysis. A getMutex function was applied, with a binary matrix of the presence/absence of truncal driver gene mutations and a binary matrix of truncal SCNA alterations (driver gene amplifications, chromosome arm gains, and arm loss/LOH) provided as input data, for the low/mid-grade predominant and high-grade predominant tumours separately. A getMutexAB function was also run, with binary matrices of the presence/absence of truncal driver gene mutations and truncal SCNA alterations provided as input data. The outputs from getMutex and getMutexAB functions were combined, and the probabilities of mutual exclusivity and co-occurrence were adjusted for multiple comparisons using the BH method. To focus on the mutually exclusive or co-occurring truncal events specific to predominantly high-grade or low/mid-grade tumours, the events with unadjusted *P* value < 0.05 in both predominantly high-grade and low/mid-grade tumours were filtered out. Analyses were conducted using R 4.0.0 (R Foundation for Statistical Computing, Vienna, Austria). The same analyses were conducted including only truncal alterations observed in at least 10% of the cohort or using Sequenza^18^ as an orthogonal tool for SCNA profiling.

### GISTIC2.0 peak identification for tumour regions with solid pattern

GISTIC2.0^21^ takes as input a copy number profile across the genome from one sample per tumour. To investigate genomic regions of recurrent gains and amplifications associated with solid pattern, the copy number profiles from all solid-pattern regions from the same tumour were uniformly segmented by taking minimum consistent segmentations and the single sample copy number profile for each tumour was constructed by selecting the minimum ploidy-corrected total copy number per segment across the genome. By taking the minimum ploidy-corrected total copy number per segment, a significant peak (*q* < 0.05) in chromosome 3q (chr3:131091386-191871390, 3q21.3-3q29) was inferred as a truncal focal amp/gain peak associated with the presence of the solid-pattern regions. To investigate the presence of the gain involving the focal 3q segment in each tumour region, mean ploidy-adjusted copy number (CN) of the focal 3q segment was calculated and log2 transformed. When all tumour regions harboured mean ploidy-adjusted CN > log2(2.5/2), the tumour was classified as having a truncal gain of the focal 3q segment (including chromosome 3q arm gain), and the tumour was classified as having subclonal gain when not all but at least one region harboured a gain of the segment.

### Identification of factors independently associated with tumour cell PD-L1 protein expression

To investigate whether the proportion of solid pattern per tumour is independently associated with tumour cell (cancer cell) PD-L1 protein expression (0% vs ≥1%), we performed logistic regression analysis (response variable = PD-L1 protein expression) to account for potential confounders including the amount of TILs (pathology TIL scores), truncal and subclonal neoantigen burden, and the presence of any loss of heterozygosity of human leukocyte antigen (HLA LOH) inferred using LOHHLA^23^. Variables with *P*<0.2 at univariate analysis were included for the multivariable analysis: pathology TIL scores, truncal and subclonal neoantigen burden (log10), and solid pattern %.

### Genomic distance

The genomic distance using mutations was calculated as previously described^2^. In brief, all detected mutations (SNVs and indels) present in any region of a tumour were turned into a binary matrix (1: mutation present, 0: mutation absent), in which the rows were the mutations and the columns were tumour regions. The pairwise Euclidean distance between any two tumour regions within each tumour was calculated. The genomic distance using LOH was calculated similarly. In brief, the presence of cytoband level LOH was first assigned when the copy-number status of the largest genomic segment overlapping each cytoband was LOH. Then, all copy-number states per cytoband in any region from a tumour were turned into a binary matrix (1: LOH present, 0: LOH absent), in which the rows were genomic segments (cytoband) and the columns were tumour regions, and the pairwise Euclidean distance between any two tumour regions was calculated. Mixed effects models are regression analyses for repeated data measures (e.g. where one patient provides multiple outcomes), and a linear mixed effect model was applied for the comparison of genomic distances between regional pairs with same vs different growth patterns to account for multiple regional pairs from a single tumour (response variable = genomic distance, fixed effect = growth pattern (different vs same), random effect = tumour ID), using the nlme (3.1-153) R package.

### Sequential evolution from lower-grade pattern to higher-grade pattern

#### Application of grade pattern scoring

To compare the regional growth patterns between specific groups of regions (for example, seeding vs non-seeding regions in the primary tumour, or seeding regions vs metastasis samples), growth pattern was transformed into integer scores as follows: lepidic, 0 (low-grade pattern); papillary and acinar, 1 (mid-grade pattern); and cribriform, micropapillary, and solid, 3 (high-grade pattern) and then the mean of regional pattern grade scores calculated for each regional group within each tumour.

#### Ancestor-descendant-like relation inference

Within the primary tumours, regional pairs having ancestor-descendant-like relations were investigated under the assumption that although a tumour region has not directly evolved from another region, there might be a regional pair in which one region is harbouring a common ancestral-like clone and another region is harbouring a descendant-like clone of the common ancestral clone. Assignments of ancestor-like region and descendant-like region were made as follows:

1. First, primary tumour regions with purity <0.15 were removed, in case LOH calling in low purity regions was not robust;
2. Next, a LOH tree was built for all regional pairs by counting shared (=trunk) LOH and private (=branch) LOH per cytoband;
3. Based on the hypothesis that the majority of LUAD tumours have one or more truncal arm-level LOH events, and that regional pairs without any shared arm-level LOH may be due to technical errors (e.g. inappropriate ploidy/purity solutions), only LOH trees with at least one shared arm-level LOH were retained for further analysis;
4. For the remaining regional pairs and LOH trees,
  1. An *ancestor-like region*, namely, a region that harbours a clone similar to a recent common ancestral clone, was defined as a region with a private LOH branch length less than X% of the trunk length, where X=2 is used in the main text and figure.
  2. A *descendant-like region* was defined as a region with a private LOH branch length of more than Y% of the trunk length and more than one arm-level private LOH, where Y=10 is used in the main text and figure.

To test if the ancestor-descendant-like relations inferred by the LOH profile conflict with mutational profiles, dominant mutations with cancer cell fraction (CCF) ≥95% (namely, mutations shared among ≥95% cancer cells within each region) were compared between paired descendant-like regions and ancestor-like regions.

Ancestor-descendant-like regional pairs were also inferred using both LOH and mutational profiles together, applying the same method for dominant mutations (CCF ≥95%). Namely, a mutation tree (CCF ≥95%) was built for all regional pairs by counting shared mutations (=trunk) and private mutations (=branch), and the ancestor-like region was defined as a region with private mutational branch length less than 2% of the trunk length, and the descendant-like region was defined as a region with a private mutational branch length of more than 10% of the trunk length. In the combined method of LOH and mutational profiles, the regional pairs called as having ancestor-descendant-like relations by both methods were inferred as ancestor-descendant-like regional pairs. Additionally, as an orthogonal method for calling SCNA profiles, we applied Sequenza^18^, and otherwise, the same definition for inferring ancestor-descendant-like relations.

#### Permutation test

To test the enrichment of higher grade patterns in the descendant-like regions and the enrichment of higher mutational burden (CCF≥95%) in the descendant-like regions (namely, lower-to-higher (upward) transition), permutation tests were applied to obtain empirical P values using the Monte-Carlo procedure^22^. Firstly, the number of ancestor-descendant-like regional pairs with the upward transition was counted (= observed frequency). Next, for each permutation, regional growth patterns and regional mutational burdens were randomised within each tumour and the frequency of upward transition was compared against the observed frequency. Finally, the empirical *P* value was calculated by Equation 1:

[Equation 1] P=(r+1)/(n+1)

where r is the number of permutations that produced a higher frequency of upward transition compared with the observed frequency and n is the number of permutations.

### Histological factors associated with tumour cell spread through air spaces (STAS) and pre-operative circulating tumour DNA (ctDNA) positivity

To elucidate the biological difference between STAS positivity and pre-operative ctDNA positivity, a univariable logistic regression model was applied. For the response variable, either STAS positivity or preoperative ctDNA positivity was used. For the explanatory variable, each of the following histological variables was included in the model: the presence of each growth pattern, mitotic index, nuclear grade, Ki-67 fraction of tumour cells, type of tumour (IMA or not), presence of necrosis, lymphovascular invasion, visceral pleural invasion, and pathological tumour size. *P* values of ANOVA in each univariable model were adjusted for multiple comparisons by the BH method.

### Gene set enrichment analyses

The median value of the variant stabilising transformed count of each gene was calculated per tumour, and gene set enrichment analyses (GSEA) were performed using the fgseaLabel function (fgsea (1.12.0) R package) to compare differentially expressed gene sets between high-grade predominant tumours and low/mid-grade predominant tumours, and between STAS positive and STAS negative tumours using the Molecular Signatures Database (MSigDB) hallmark gene sets^24^. Multiple comparisons were adjusted by the BH method and gene sets with *q* < 0.25 were determined to be significantly enriched as described elsewhere^25^.

### Enrichment analysis of G2M checkpoint gene SCNA

Mean ploidy-adjusted copy number of each gene was calculated per tumour region and was compared between predominantly high-grade tumours and low/mid-grade tumours using a linear mixed effect model by setting the ploidy adjusted copy number of each gene as a response variable, predominance of growth pattern (high vs low/mid) as a fixed effect variable, and tumour ID as a random effect using the nlme (3.1-153) R package. ANOVA P values were adjusted for multiple comparisons by the BH method and the genes with *q* < 0.05 were determined to have significantly variant copy numbers between predominantly high- and low/mid-grade tumours. Among the 13728 genes tested, 1032 were significantly gained and 749 were lost in predominantly high-grade tumours. Over-representation of SCNA of G2M checkpoint genes^24^ was tested by chi-square goodness of fit test.

### Survival analysis (TRACERx cohort)

Disease-free survival (DFS) was defined as the period from the date of registration to the time of radiological confirmation of the recurrence of the primary tumour registered for the TRACERx or the time of death by any cause. During the follow-up, three patients (CRUK0512, CRUK0428, and CRUK0511) developed new primary cancer and subsequent recurrence from either the first primary lung cancer or the new primary cancer diagnosed during the follow-up. These cases were censored at the time of the diagnosis of new primary cancer for DFS analysis, due to the uncertainty of the origin of the third tumour. As for the patients who harboured synchronous multiple primary lung cancers, when associating genomic/pathologic data from the tumours with patient level clinical information, we used only data from the tumour of the highest pathological TMN stage. Hazard ratios and *P* values were calculated using the coxph function (survival (3.3.1) R package), through multivariable Cox regression analyses, adjusted for age, stage, pack-years, surgery type, and adjuvant therapy. Kaplan–Meier plots were generated using ggsurvplot function (survminer (0.4.9) R package). Intra-thoracic relapse was defined as any relapse found within the thoracic cavity including mediastinum and parietal pleura but not ribs. Extra-thoracic relapse was defined as any relapse found outside the thoracic cavity, including ribs and axillary lymph nodes. To estimate the relapse-site specific (subdistribution) hazard ratio, the Fine-Gray competing risk regression model was applied using cmprsk (2.2-11) R package and adjusted for age, stage, pack-years, surgery type, and adjuvant therapy. In this analysis, relapses at the specific site (intra-thoracic-only or extra-thoracic with or without intra-thoracic) are counted as events, and relapses at other sites or non-lung cancer deaths are treated as competing events. Relapsed cases with uncertain sites and/or uncertain origins were excluded from relapse site-specific analysis.

### Survival analysis (Memorial Sloan Kettering Cancer Center cohort)

#### Patient selection

From the Memorial Sloan Kettering Cancer Center Thoracic Surgery Service prospectively maintained database, 712 patients who had undergone surgical resection for p-stage (TNM staging, version 7) IB to IIIA primary lung adenocarcinoma from January 2006 to December 2014 were identified. Exclusion criteria were: receipt of induction therapy, non-invasive histology, and R2 resection classification.

#### Statistical analysis

The distribution of patient clinicopathologic characteristics was summarised as frequencies and percentages or medians, 25^th^, and 75^th^ percentiles for categorical and continuous factors, respectively. Disease-free survival (DFS) was calculated from the date of surgery to the date of any recurrence or death, whichever occurs first. Patients were otherwise censored on the date of the last follow-up. DFS was estimated using the Kaplan-Meier method and compared between groups using the log-rank test. The relationships between factors of interest (STAS status, necrosis status, and the combination of the two) and DFS were quantified using multivariable Cox proportional hazards models, controlling for age at surgery, pathologic stage (1, 2, 3), pack-years, surgery type (sublobar vs lobar or greater), and any adjuvant therapy. All statistical tests were two-sided and P<0.05 was considered statistically significant. Analyses were conducted using Stata 15.1 (StataCorp, College Station, TX) and R 4.1.2 (R Foundation for Statistical Computing, Vienna, Austria).

### Statistical analysis

All statistical tests were performed in R (version 3.6.1 unless specified otherwise). Tests involving correlations were performed using cor.test with Spearman’s method. Tests involving comparisons of distributions were performed using Wilcoxon rank sum test (or Wilcoxon signed-rank test for paired analysis) or linear mixed effect regression analysis as stated, using wilcox.test or lme (nlme (3.1-153) R package) functions, respectively. *P* values were adjusted for multiple comparisons using the BH method unless otherwise stated, and reported as *q* values. All statistical tests were two-sided unless specified otherwise, and the numbers of data points included are plotted and/or annotated in the corresponding figures or figure legends. Plotting was done by using ggplot2 (3.3.5) and ComplexHeatmap (2.2.0) R packages.

## Extended Data

## Supplementary Material

Supplementary Tables

## Data Availability

All code to reproduce figures will be made available at publication or upon request from reviewers.
